# An open-source, automated, and cost-effective platform for COVID-19 diagnosis and rapid portable genomic surveillance using nanopore sequencing

**DOI:** 10.1038/s41598-023-47190-w

**Published:** 2023-11-21

**Authors:** Gerardo Ramos-Mandujano, Raik Grünberg, Yingzi Zhang, Chongwei Bi, Francisco J. Guzmán-Vega, Muhammad Shuaib, Rodion V. Gorchakov, Jinna Xu, Muhammad Tehseen, Masateru Takahashi, Etsuko Takahashi, Ashraf Dada, Adeel Nazir Ahmad, Samir M. Hamdan, Arnab Pain, Stefan T. Arold, Mo Li

**Affiliations:** 1 Stem Cell and Regeneration Laboratory, Bioscience Program, Biological and Environmental Science and Engineering Division (BESE), King Abdullah University of Science and Technology (KAUST), 23955-6900 Thuwal, Kingdom of Saudi Arabia; 2 Structural Biology and Engineering, Computational Biology Research Center. Biological and Environmental Science and Engineering Division (BESE), King Abdullah University of Science and Technology (KAUST), 23955-6900 Thuwal, Kingdom of Saudi Arabia; 3 Pathogen Genomics Laboratory, Bioscience Program, Biological and Environmental Science and Engineering (BESE), King Abdullah University of Science and Technology (KAUST), 23955-6900 Thuwal, Kingdom of Saudi Arabia; 4 Health, Safety and Environment Department, King Abdullah University of Science and Technology (KAUST), 23955-6900 Thuwal, Kingdom of Saudi Arabia; 5 Laboratory of DNA Replication and Recombination, Bioscience Program, Biological and Environmental Science and Engineering Division (BESE), King Abdullah University of Science and Technology (KAUST), 23955-6900 Thuwal, Kingdom of Saudi Arabia; 6 Department of Pathology and Laboratory Medicine, King Faisal Specialist Hospital and Research Center, Jeddah, Kingdom of Saudi Arabia; 7 College of Medicine, Al Faisal University, Riyadh, Kingdom of Saudi Arabia; 8 KAUST Health, King Abdullah University of Science and Technology (KAUST), 23955-6900 Thuwal, Kingdom of Saudi Arabia; 9 Bioengineering Program, Biological and Environmental Science and Engineering Division (BESE), King Abdullah University of Science and Technology (KAUST), 23955-6900 Thuwal, Kingdom of Saudi Arabia

**Keywords:** Infectious-disease diagnostics, Pathogens, Genome informatics, Next-generation sequencing, Biological techniques, Genomic analysis, Infectious diseases, Viral infection

## Abstract

The COVID-19 pandemic, caused by SARS-CoV-2, has emphasized the necessity for scalable diagnostic workflows using locally produced reagents and basic laboratory equipment with minimal dependence on global supply chains. We introduce an open-source automated platform for high-throughput RNA extraction and pathogen diagnosis, which uses reagents almost entirely produced in-house. This platform integrates our methods for self-manufacturing magnetic nanoparticles and qRT-PCR reagents-both of which have received regulatory approval for clinical use–with an in-house, open-source robotic extraction protocol. It also incorporates our "Nanopore Sequencing of Isothermal Rapid Viral Amplification for Near Real-time Analysis" (NIRVANA) technology, designed for tracking SARS-CoV-2 mutations and variants. The platform exhibits high reproducibility and consistency without cross-contamination, and its limit of detection, sensitivity, and specificity are comparable to commercial assays. Automated NIRVANA effectively identifies circulating SARS-CoV-2 variants. Our in-house, cost-effective reagents, automated diagnostic workflows, and portable genomic surveillance strategies provide a scalable and rapid solution for COVID-19 diagnosis and variant tracking, essential for current and future pandemic responses.

## Introduction

Rapid, sensitive diagnosis of infection and genetic surveillance of the pathogen are critical for effective control and monitoring of infectious diseases^1^. The COVID-19 pandemic demonstrated how a rapid increase in clinical and community testing demands can quickly overwhelm the locally available testing capability. Most diagnostic workflows depend on specialized instruments, and their exclusive reagent supplies quickly become bottlenecks, creating an urgent need for approaches that increase testing capacity without depending on expensive and proprietary consumables^2^. This need has encouraged academic and commercial laboratories to develop in-house and open-source workflows for high-throughput COVID-19 testing, as well as a roadmap for tracking SARS-CoV-2 variants^3^. Beyond COVID-19, such approaches would enable a resilient response to future viral pandemics, especially in countries with limited resources or during times when global supply chains are under strain.

A high-throughput workflow for diagnosing RNA viral pathogens requires the provision of scalable RNA extraction and qRT-PCR kits^4^. RNA extraction based on solid-phase reversible immobilization using coated magnetic beads is inherently scalable and can be prepared in most laboratories using inexpensive reagents^5^. However, few “home-made” protocols have been validated in clinical laboratories and received governmental regulatory approval for COVID-19 diagnosis. We previously reported the magnetic-nanoparticle-aided viral RNA isolation from contagious samples (MAVRICS) protocol, built upon readily available reagents and easily assembled in any basically equipped laboratory^6^. qRT-PCR is the gold standard of identifying the presence of viral pathogens. However, high costs and unreliable supply chains may limit the applicability of such methods during a pandemic. Ideally, diagnostic reagents and scalable workflows should therefore be sourced locally, in adherence to globally shared protocols, and without being hindered by patents or commercial supplies. Importantly, few self-manufacturing methods for either RNA extraction or qRT-PCR kits have been rigorously tested in clinical laboratories to obtain approval from relevant local authorities. There have been no prior reports of in-house or open-source workflows tailored for rapid and high-throughput diagnosis and genome surveillance of SARS-CoV-2 or other viral pathogens.

Genomic surveillance is critical for early detection of mutations, monitoring of viral evolution, and assessing circulating variants^7,8^. However, it requires complex workflows that are separated from those for diagnosis and rely on highly specialized facilities, personnel, and reagents. We have recently integrated diagnosis and genomic surveillance workflows in a sequencing-based method called “Nanopore sequencing of Isothermal Rapid Viral Amplification for Near real-time Analysis” (NIRVANA) that monitors mutations of viral pathogens in real-time in a high-throughput manner^9,10^. Moreover, NIRVANA protocol uses isothermal recombinase polymerase amplification (RPA) that can be performed in a simple heating block, and all materials required for sequencing fit into a briefcase. Such portability enables on-site analysis in remote locations, swift deployment to outbreak hotspots, and accessible monitoring in regions with limited laboratory infrastructure, making it an invaluable asset in global health emergencies where rapid response is crucial.

In this study, we report an open-source workflow for in-house, scalable robotic RNA extraction, patent-free qRT-PCR-based diagnosis, and genome surveillance of SARS-CoV-2. The self-manufacturing protocols of our in-house RNA extraction and qRT-PCR kits were tested in clinical laboratories to meet the safety, efficacy, and quality standards required by the Saudi Food and Drug Authority (SFDA) for use in the clinical laboratories at King Abdullah University of Science and Technology (KAUST). Our workflow addresses the current needs of SARS-CoV-2 surveillance and can be deployed in future pandemics.

## Results

### Evaluating MAVRICS in clinical diagnosis for regulatory approval

**Figure 1:**
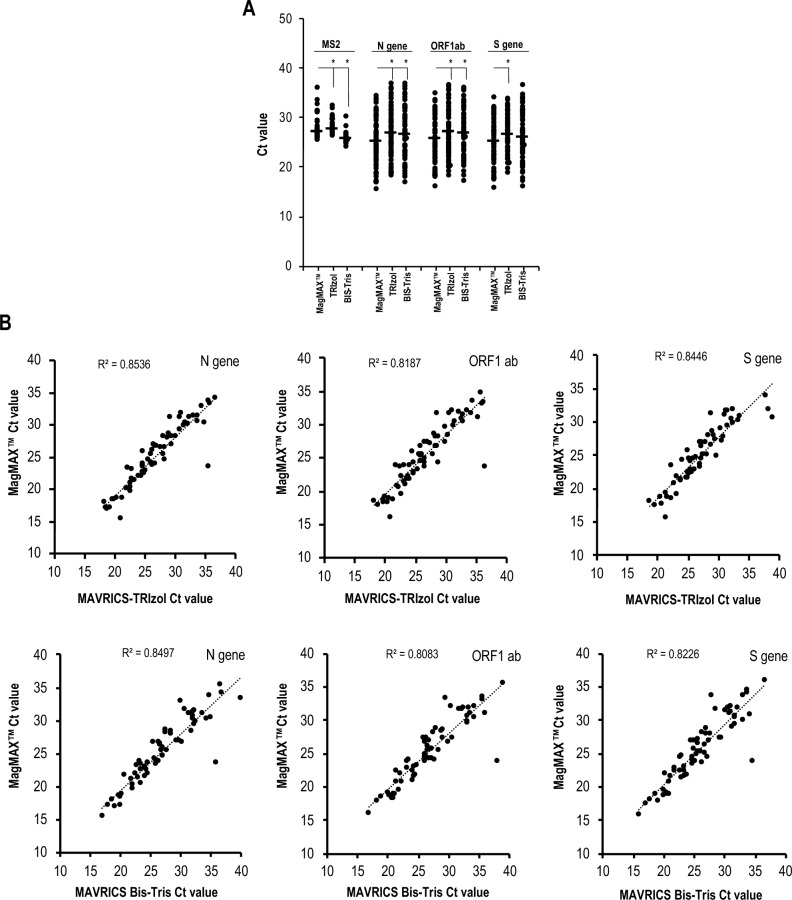
Clinical validation of MAVRICS protocols. RNA from 94 clinical samples collected in viral transport medium (VTM) was extracted in parallel using MAVRICS TRIzol, MAVRICS Bis–Tris, and MagMAX™ protocols. For SARS-CoV-2 diagnosis, SARS-CoV-2 ORF 1ab, S gene, and N gene and the internal control MS2 were detected by TaqPath™ COVID-19 Combo Kit. (**A)** Ct value (mean ± S.D.) for the targets in the three protocols, *p < 0.01 (paired Student’s t-test). (**B)** Correlation of Ct values of SARS-CoV-2 targets between tested protocols and corresponding Pearson correlation coefficient (r). The input sample was 200µl for MAVRICS-BisTris and MagMAX™ protocols and 100 µl for the MAVRICS-TRIzol protocol. The criteria for amplification curve, Ct value for each target, and SARS-CoV-2 sample detection were according to the TaqPath™ kit as described in "Methods".

The in-house produced components of the MAVRICS RNA extraction protocol^6^, consisting of silica magnetic nanoparticles (SiMNP) and buffers, were efficiently and reproducibly prepared using basic lab equipment (Supplementary Fig. 1). To validate the MAVRICS protocol for clinical diagnosis, we compared it to the commercial MagMAX™ Nucleic Acid Isolation Kit (ThermoFisher Scientific, A48383) using 94 clinical samples stored in viral transport medium (VTM). We performed RNA extraction using two versions of the MAVRICS protocol (see "Methods" and Supplementary Material 1A-C). A multiplex COVID-19 RT-PCR assay (TaqPath™ ThermoFisher Scientific, A48067) was used to detect the N gene, ORF 1, and S gene of SARS-CoV-2 and the internal control (MS2). In an RT-PCR test, a qualitative indication of the amount of viral RNA present is inferred by the cycle threshold (Ct), with a lower Ct representing higher viral RNA load. Following the manufacturer instructions, a target is interpreted as present if Ct ≤ 37, and a sample is considered positive if at least 2 out of 3 targets are present. If only 1 out of 3 targets is present, the result is inconclusive ("Methods" and Supplementary Material 2). Assays using RNA extracted with the MagMAX™ kit identified 66 samples as positive, 26 as negative, and 2 as inconclusive. Given that samples were no longer available for retesting, inconclusive samples were excluded from sensitivity and specificity analyses. When the same samples were extracted using the MAVRICS-TRIzol RNA protocol, one sample previously identified as positive by MagMAX™ was found to be negative, and five samples previously identified as negative by MagMAX™ were found to be positive. For the MAVRICS-Bis–Tris protocol, all positive samples were concordant with the MagMAX™ protocol, while six MagMAX™ negative samples were flagged as positive, and one negative sample was deemed inconclusive. These results translated to a sensitivity of 98.5% and a specificity of 80.8% for MAVRICS-TRIzol, and to a sensitivity of 100% and a specificity of 76.0% for MAVRICS-Bis–Tris. Although the Ct values produced by the MagMax™ kit and the two MAVRICS versions for the targets showed significant differences (range: 0.28 to 2 Ct units, Fig. 1A), they demonstrated high levels of correlation (Fig. 1B). The MAVRICS protocols met the safety, efficacy, and quality standards required by the SFDA for use in the clinical laboratories at KAUST.

### Robotic RNA extraction

We then automated the original MAVRICS-TRIzol protocol on a Tecan Freedom EVO 200 liquid handling robot in 96-well format to work with the in-house Rapid Research Response Team one-step qRT-PCR (R3T qRT-PCR)^11^using the 2019-nCoV CDC EUA kit (nCoV_N1, nCoV_N2 and RNase P (IDT Cat. No. 10006770)).The R3T qRT-PCR assay showed a similar absolute detection limit as the TaqPath™ kit using synthetic SARS-CoV-2 RNA as input (Supplementary Fig. 2).

A robotic RNA extraction script was developed from scratch on an existing Tecan Evo hardware platform with a 96-channel multi-channel aspirator (MCA) for rapid parallel execution. This liquid handling platform was introduced 20 years ago and remains widely available in both industrial and academic laboratories. It can be acquired at a reasonable cost (~ 35,000 USD pre-owned). To support the processing of fewer than 96 samples, the script can address anywhere from two to all 12 columns of the microplate, thereby minimizing consumption of tips and reagents. We automated MCA tip tracking and handling, so that the main script only has to request a given number of new “tip columns”. Auxiliary sub-programs then fetch columns from the next available position in a source plate and rotate in new tip racks as required, a feature absent in the original Evoware program suite. Critical parameters that needed optimization included buffer volumes, deepwell plate geometries, magnet holders, and shaking and heating durations (Supplementary Material 1D). For instance, efficient SiMNP mixing and recovery necessitated round-bottom wells as beads were otherwise not easily resuspended by shaking. A strong “striped” magnet outperformed a “magnet pin” holder for the formation of beads pellets that could be separated from the supernatant also in deepwell plates. Pipetting speed and tip-touch options were varied to avoid drops hanging and falling off pipette tips. All plates were covered with universal lids and automatically uncovered only for pipetting steps to reduce the risk of cross-contamination. All Evoware scripts, robot configuration, and demonstration videos of the workflow can be found at: https://github.com/strubelab/roboRNA.

**Figure 2:**
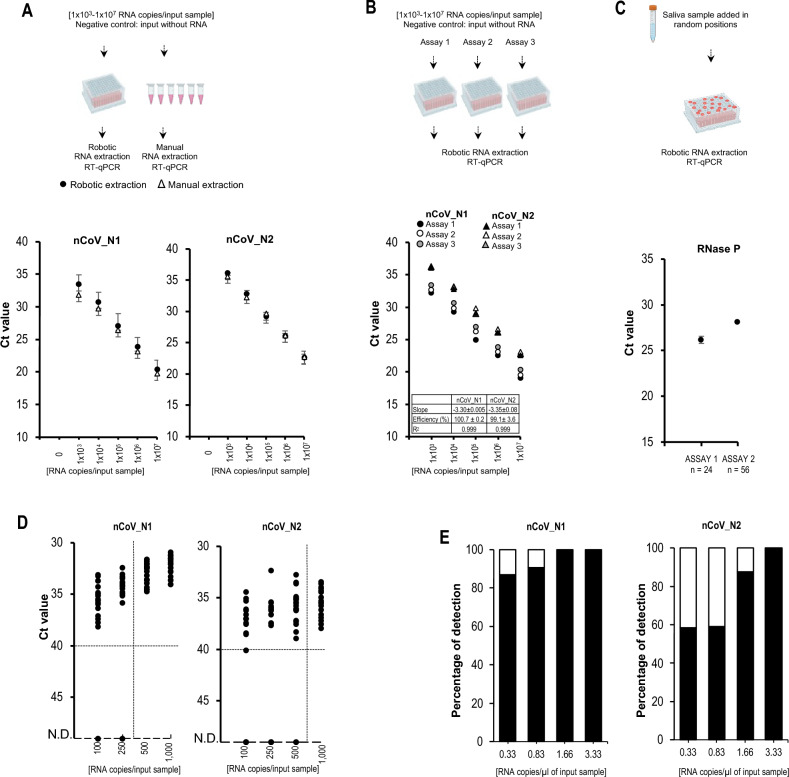
Validation and limit of detection (LoD) of robotic MAVRICS protocol. (**A**) Manual and robotic extraction were done using the same stock of input samples [1 × 10^3^ to 1 × 10^7^ RNA copies/input sample] and analyzed by the 2019-nCoV nCoV_N1 and nCoV_N2 RT-qPCR assays. No significant difference was found (Student’s t-test). (**B**) Three independent robotic RNA extractions of samples with various RNA copy numbers were completed, and the presence of nCoV_N1 and nCoV_N2 targets were determined by RT-qPCR. The slope (mean ± SD), qPCR efficiency (mean ± SD), and correlation coefficient (r^2^) were calculated. (**C**) In two independent experiments, TRIzol was added to a saliva sample that was then plated randomly in 24 (assay 1) or 56 (assays 2) wells in the input plate, followed by robotic extraction and RT-qPCR analysis of RNase P. (**D**, **E**) To determine the Limit of Detection (LoD) RNA was extracted using the robotic MAVRICS protocol from contrived SARS-CoV-2 samples [100 to 1, 000 RNA copies/input sample], and one step RT-qPCR was performed with the in-house kit R3T qRT-PCR and the probes nCoV_N1, and nCoV_N2. D. Ct values and E. Black bars represent the percentage of detection of nCoV_N1 (left) and nCoV-N2 (right) in replicates of different concentrations.

Automated robotic extraction was assessed using synthetic SARS-CoV-2 RNA samples and the in-house R3T qRT-PCR kit using the 2019-nCoV CDC EUA kit (nCoV_N1, nCoV_N2 and RNase P). The Ct values from both automated and manual RNA extraction were comparable at all RNA concentrations tested (Fig. 2A). To evaluate reproducibility three independent automated extractions were performed, which showed high PCR efficiencies and correlation coefficients > 0.999 (Fig. 2B). Cross-contamination was absent since neither nCoV_N1 nor nCoV_N2 was detected in samples without synthetic RNA distributed randomly across the input plate (Fig. 2B and data not shown). In addition, a SARS-CoV-2 negative saliva sample was added to random positions of the input plate in two independent runs. The RNase P Ct values of the samples on the same plate showed low standard deviations, indicating consistent RNA extraction irrespective of sample position in the plate (Fig. 2C). These data validated that automated RNA extraction is reliable and devoid of cross-contaminations.

For molecular diagnosis, a crucial parameter for analytic sensitivity is the limit of detection (LoD), defined as the lowest concentration of the target that can be detected in ≥ 95% of replicate measurements^12^. We therefore determined the LoD of the robotic MAVRICS and R3T RT-qPCR workflow with a minimum of 20 replicates per concentration. Viral RNA was detected in all concentrations tested, even as low as 0.33 RNA copies/µl (Fig. 2D and E). However, the defined LoD (vertical dashed lines, Fig. 2D) was 1.66 and 3.33 RNA copies/µl for nCoV_N1 and nCoV_N2, respectively (Fig. 2E).

**Figure 3:**
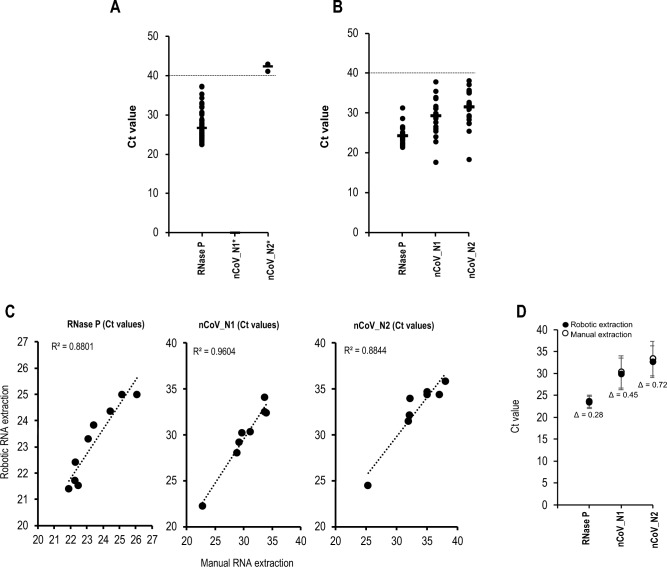
Robotic extraction from swab clinical samples. Robotic MAVRICS RNA extraction was performed in clinical samples using a Tecan EVO-200 system, one-step RT-qPCR was performed with R3T RT-qPCR system and the nCoV_N1, nCoV_N2 and RNase P assays. (**A** and **B**) Ct values for RNase P, nCoV_N1 and nCoV_N2 obtained from the samples categorized as negative (**A**) and positive (**B**) for SARS-CoV-2. *: Ct value undetermined due to no amplification for most samples. (**C** and **D**) Correlation (**C**) and difference (**D**) of Ct values between robotic and manual extraction from eight positives samples for RNase P, nCoV_N1 and nCoV_N2. No significant difference was found (Student’s t-test).

We obtained 106 samples collected in TRIzol reagent with prior clinical diagnoses (29 SARS-CoV-2 positive, 67 negative, and 10 inconclusive or invalid (Supplementary Material 3). RNA was extracted using robotic MAVRICS and COVID-19 was diagnosed using the R3T qRT-PCR assay (Supplementary Material 3). The outcomes were 23 positive, 72 negative, and 11 inconclusive or invalid samples. Comparing the reference results with ours yielded a diagnostic congruence of 82.1% (87/106) (Supplementary Material 3). In our workflow, negative samples exhibited Ct values for RNase *P* ranging from 22.42 to 37.3, with nCoV_N1 and nCoV_N2 being either undetectable or displaying a Ct value > 40 (the 2019-nCoV assay sets the Ct value threshold for detection at ≤ 40, as illustrated in Fig. 3A). The Ct values of positive samples ranged from 21.3 to 31.27 for RNase P, 23.9 to 37.7 for nCoV_N1, and 27.31 to 38.0 for nCoV_N2 (Fig. 3B). For a direct assessment of the real-world performance of the robotic RNA extraction, we processed eight positive samples in parallel with both robotic and manual RNA extractions. All samples were deemed positive by both methods with highly consistent Ct values (Fig. 3C and D).

### Variant tracking by NIRVANA

**Figure 4:**
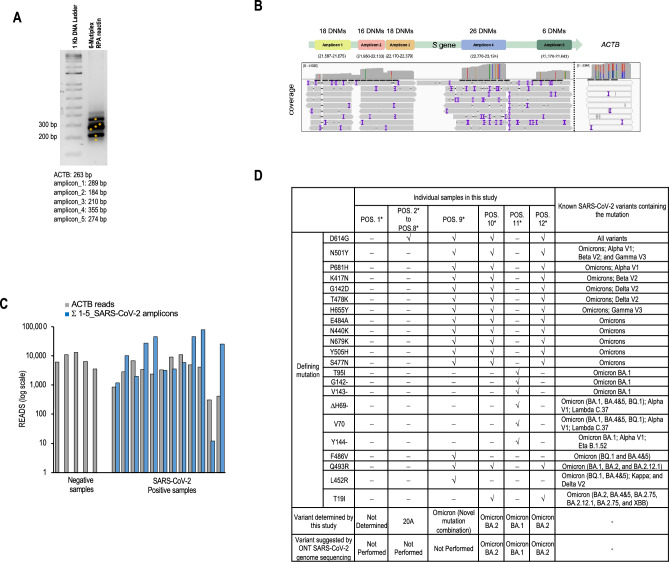
SARS-CoV-2 diagnosis and variant identification by the NIRVANA protocol. RNA samples were subjected to reverse transcription, followed by multiplex RPA to amplify five regions of the SARS-CoV-2 genome and the internal control ACTB. For sequencing, the amplicons were purified and prepared for nanopore sequencing using an optimized barcoding library preparation protocol. Sequencing was performed in the pocket-sized Nanopore MinION sequencer and sequencing results were analyzed by the algorithm termed RTNano on the fly. (**A**) Representative agarose gel electrophoresis result of multiplex RPA products from one positive sample. (**B**) Diagram of the genomic location of RPA amplicons and the number of SARS-CoV-2 DNMs (defining nucleotide mutations) covered by them. The IGV track shows sequencing coverage of the SARS-CoV-2 amplicons and the internal control ACTB amplicon in one sample. C. Read counts of the five SARS-CoV-2 amplicons and ACTB for the sequenced samples. D. Detected defining mutations and identified variants in the positive samples. POS. 1–12 are abbreviations of individual positive samples. The SARS-CoV-2 genome sequencing data of samples POS. 10, 11, and 12 have been deposited to the GISAID with IDs EPI_ISL_15827169 (POS. 10), EPI_ISL_15826832 (POS. 11), and EPI_ISL_15826930 (POS. 12). The original gels related to (**A**) are presented in Supplementary Material 7.

Variant tracking was conducted using NIRVANA^9^ to target mutation hotspots of the SARS-CoV-2 spike gene. RPA primer pairs were designed (see "Methods") to encompass the current SARS-CoV-2 variants listed on covariants.org^13^. We screened for RPA primers using a positive patient sample (CoV_N1 Ct = 25) and selected five primer pairs that showed robust and specific products when used separately (Supplementary Fig. 3) and when combined in a multiplex RPA reaction that included the internal control ACTB (Fig. 4A). The targeted SARS-CoV-2 genomic regions contain 84 mutations that are widely distributed in variants being monitored and the variants of concern as certified by the CDC (Centers for Disease Control and Prevention) (Supplementary Material 4 and Fig. 4B). The multiplex reaction was prepared and sequenced using an R9.4.1 flow cell on a Nanopore MinION sequencer. Hundreds of reads were aligned to each of the five targeted mutation hotspots in the SARS-CoV-2 reference genome, confirming the effectiveness of the multiplex RPA reaction and the ensuing NIRVANA workflow (Fig. 4B).

Seventeen RNA samples, previously extracted using the robotic MAVRICS protocol and diagnosed as either SARS-CoV-2 positive (12 samples, named as POS. 1 to POS. 12) or negative (5 samples, named as NEG. 1 to NEG. 5), were processed for multiplex nanopore sequencing using the Oxford Nanopore Native Barcodes (see "Methods"). To evaluate cross-contamination among samples in a 96-well plate, and establish a threshold read number for amplicon alignment, we added negative controls in the robotic extraction (NTC-R) and no-template controls (NTCs) for the reverse transcription (NTC-RT) and RPA reactions (NTC-RPA) (Supplementary Material 5). Three libraries, comprising samples POS.1–8, samples POS.5 and NEG. 1–3, and samples POS. 9–12 and NEG. 4–5, respectively, were each subjected to a sequencing run. Two, seven, and four NTCs were included in each sequencing run to ensure quality control. The runs yielded 511,359 reads, 485,811 reads, and 763,566 reads, respectively. The NTCs presented up to 9 reads for SARS-CoV-2 or ACTB amplicons, representing 0.006% of the total reads. We found that the total number of reads per amplicon correlated positively (r^2^ = 0.63, Pearson’s *r* = 0.79) with the total number of reads aligned in the NTCs (Supplementary Material 5), suggesting that reads in NTC samples were due to misclassification of sample barcode possibly caused by basecalling errors in the barcodes^9^. We set two times the maximal read count in the NTC as the background alignment threshold; the amplicons that had reads below this threshold were not analysed further (Supplementary Material 5).

In the five SARS-CoV-2-negative samples, between 5773 to 12,196 reads aligned to the ACTB target sequence, and the reads for all SARS-CoV-2 amplicons were below the alignment threshold (Fig. 4C and Supplementary Material 5*).* In the twelve SARS-CoV-2-positive samples, more than 300 reads per sample were identified for ACTB (Fig. 4C), with a minimum of two SARS-CoV-2 amplicons aligned in each sample. All other positive samples had between 1,187 and 76,860 reads for all SARS-CoV-2 amplicons except for POS. 11, which had 12 reads (Supplemental Material 5 and Fig. 4C). When one positive sample was subjected to the NIRVANA workflow in two independent trials (POS. 5A and 5B in Supplementary material 5), both the number and percentage of reads for each amplicon displayed high concordance between trials (r^2^ 0.833 and Pearson coefficient 0.913 for both measurements), highlighting the reproducibility of the workflow. Among the twelve positive samples, there was a strong correlation (r^2^ = 0.739 and 0.746) between the Ct values and the total reads in the SARS-CoV-2 amplicons, suggesting that the SARS-CoV-2 read count faithfully reported the viral load (Supplementary Material 6). For samples POS. 1–8 sequenced using an R9.4.1 flow cell, the Pearson correlation coefficients between Ct and read numbers were 0.70 and 0.71 in two independent trials, consistently affirming the reliability of SARS-CoV-2 read counts in reporting the viral load. For samples POS. 9–12 sequenced using an R10.4.1 flow cell, the sample size was insufficient for correlation analysis.

Variant detection was performed by using an updated version of our previously developed real-time nanopore sequencing monitor (RTNano)^10,14^. Twenty-two defining mutations (see "Methods" and Supplementary Material 4) were distributed in 11 of the 12 positive samples (Fig. 4D). Seven samples (POS. 2 to POS. 8) contained only the D614G mutation (in the surveyed genomic regions), which had occurred early in the pandemic and defined the clade 20A (https://nextstrain.org/). POS. 1 could not be classified confidently as either wild type or clade 20A because the read count covering the D614 position was below the threshold (Fig. 4D, Supplementary Material 5). POS. 9, POS. 10, and POS. 12 had 15, 14, and 14 mutations, respectively. These samples contained mutations D614G, N501Y, P681H, K417N, G142D, T478K, and H655Y which are present in all Omicron variants and in Alpha V1, Beta V2, Delta, and Gamma V3 variants. In addition, these samples had mutations present only in the Omicron variants (i.e., E484A, N440K, N679K, Y505H, and S477N), and were therefore defined as Omicron. POS.10 and POS. 12 also had mutations (Q493R and T19I) typical of different Omicron sub-lineages and could be classified as Omicron BA.2. POS. 9 had two mutations (F486V and K417N) exclusive to Omicron variants BA.4&5 and BQ1, and one mutation (Q493R) exclusive to Omicron variants BA.1, BA.2, and BA2.12.1, which suggests sample POS. 9 was infected with an Omicron variant with a previously unreported combination of mutations (Fig. 4D). POS. 11 had the mutations T95I, G142-, V143-, ΔH69, V70, and Y144-, and was categorized as the Omicron BA.1 variant. POS. 10, POS. 11, and POS. 12 had also undergone SARS-CoV-2 genome sequencing using the Oxford Nanopore Technologies (ONT) platform, adhering to the protocol by Freed et al. (https://www.protocols.io/view/sars-cov2-genome-sequencing-protocol-1200bp-amplic-rm7vz8q64vx1/v6), except that the artic primer set V4.1 (https://github.com/joshquick/artic-ncov2019/tree/master/primer_schemes/nCoV-2019/V3) was used. Sequenced data were analyzed using the wf-artic workflow from Epi2me-labs (https://github.com/epi2me-labs/wf-artic), which fully confirmed our variant tracking results (Fig. 4D).

## Discussion

**Figure 5:**
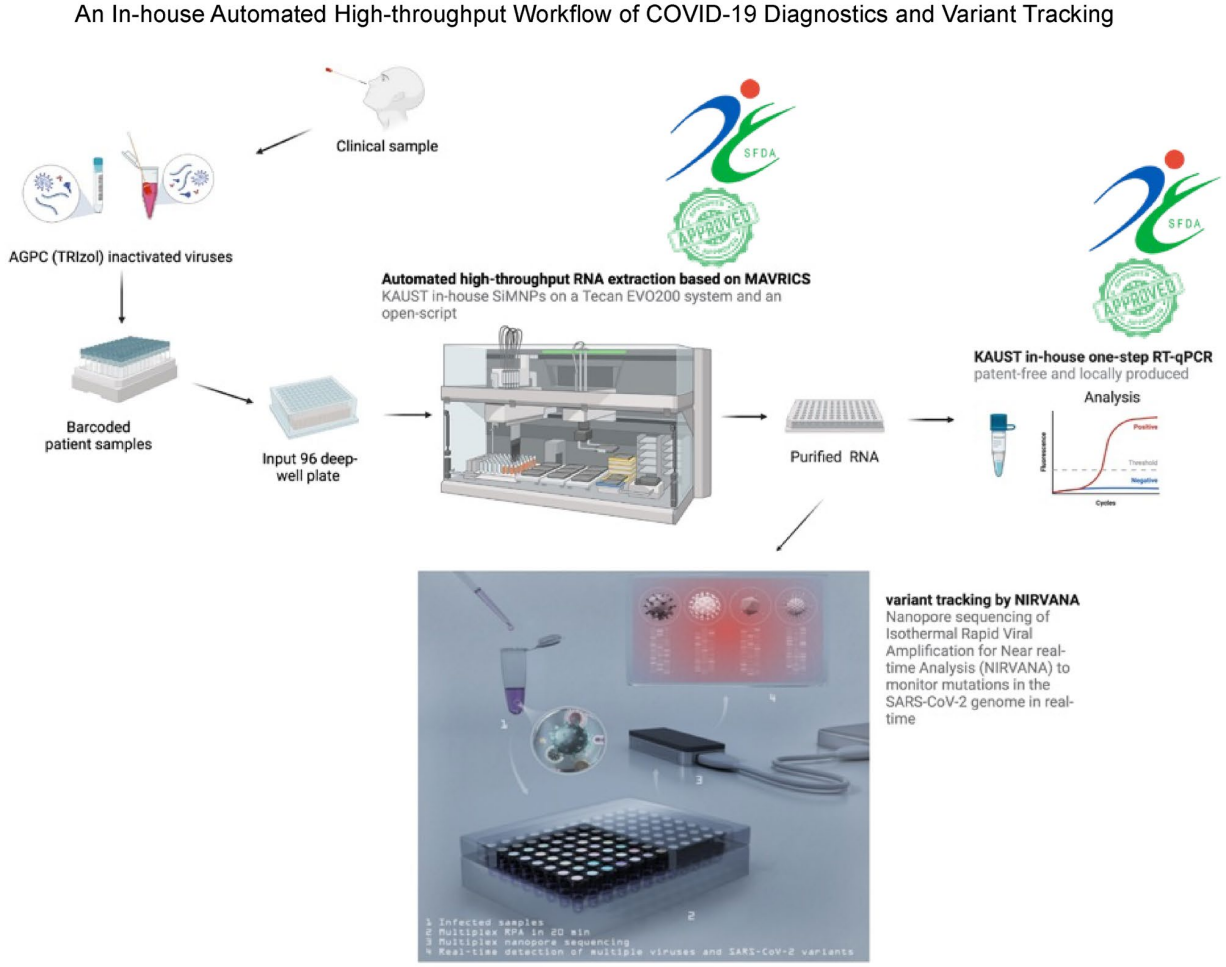
An In-house Automated High-throughput Workflow of COVID-19 Diagnostics and Variant Tracking. Clinical samples are collected and inactivated in acid guanidinium thiocyanate-phenol chloroform (AGPC) reagent as TRIzol, and sample identification is facilitated using a 2D barcoded system. The samples are then aliquoted into input 96 deep well/plates, and the robotic MAVRICS extraction is conducted using an open-script on a Tecan Evo system with in-house produced silica magnetic nanoparticles (SiMNP) and buffers described in the SFDA-approved MAVRICS protocol. The RNA obtained is tested for SARS-CoV-2 using the SFDA-approved in-house R3T (Rapid Research Response Team) one-step qRT-PCR (R3T qRT-PCR). To monitor SARS-CoV-2 mutations and track variants in real-time, RNA samples are prepared and sequenced using the Nanopore sequencing of Isothermal Rapid Viral Amplification for Near-real-time Analysis (NIRVANA) protocol.

The global health crisis triggered by SARS-CoV-2 has demonstrated the importance of population testing and molecular surveillance in managing the pandemic. However, the shortage of reagents, high cost, and the need for highly specialized infrastructure and skilled personnel hamper diagnosis and molecular surveillance in a pandemic. We have developed an open-source workflow for high-throughput testing coupled with a reliable and portable technology to track SARS-CoV-2 variants (Fig. 5).

To our knowledge, there are no previously reported in-house or open-source workflows specifically designed to diagnose SARS-CoV-2 or other viral pathogens. Although manual RNA extraction with “home-made” magnetic beads^15–18^ or with “home-made” buffers and commercial beads^19^ has been reported, existing automated protocols typically rely on commercial beads, open or proprietary robotic platforms, and patented qRT-PCR reagents^2,5,20–27^.

In the few previous studies, the analytical sensitivity, or LoD, has ranged from 1.2 to 3.04 RNA copies/µl^11,25,27^, with a sensitivity between 87.5% and 92.8% and specificity between 83 and 100%^23,28^. Virological tests and approved qRT-PCR kits possess an LoD ranging from 0.1 to 150 RNA copies/µl, with an accepted sensitivity and specificity bracket of 80% to 100%^12,29^.

In our study, the LoD of 1.66 copies/µl and 3.33 copies/µl are comparable to previous findings and rank among the lowest values for virology tests. Our platform demonstrates a diagnostic sensitivity of up to 100% and a specificity of up to 80.8%, indicating its robust and reliable performance for COVID-19 diagnosis. Furthermore, our automated RNA extraction method has the capacity to process one 96-well plate every 60 min, suggesting a daily capacity of ~ 2000 samples, while ensuring high reproducibility and the absence of cross-contamination.

NIRVANA is a platform based on targeted sequencing using the nanopore sequencing technology and can be utilized with portable materials and equipment^9,17,30^. The five RPA amplicons were selected to target the spike region of the viral genome that is one of the most informative regions for variant identification due to frequent mutations. Our approach identified variants in Clade 20A and Omicron variants. One sample (POS. 9) featured mutations for both Omicron variants BQ.1 or BA 4&5 (L452D and F486V) and for Omicron variants BA.1 or BA.2 (Q493R). This could be due to sample cross-contamination, co-infection, or a novel mutation combination. The sample had 17,818 reads of the amplicon_4 that contained these three mutations (Supplementary Material 5), indicating sufficient sequencing coverage. Co-infection with different variants of SARS-CoV-2 should result in heterogeneity in mapped reads, where the pattern of detected mutations is not consistent among all reads^31,32^. Co-infection or cross-contamination in POS. 9 was excluded because the same mutation pattern was found in all reads. This suggests POS. 9 contains an Omicron variant with a previously unknown combination of mutations. Considering the collection timeline of samples—early pandemic for POS. 2 to POS. 8 and early 2022 for POS. 9 to POS. 12—the identified strains align with the documented evolution of SARS-CoV-2 (https://nextstrain.org/). The specific Omicron variants or sub-variants defined by NIRVANA for samples POS. 10, POS. 11, and POS. 12 matched the whole-genome sequencing results. This indicates that the RNA extracted with the in-house reagents and robotic platform has the necessary quality for deep-sequencing and that the multiplex reaction and sequencing procedure are reliable in identifying current SARS-CoV-2 variants.

Efficient and accessible methods for diagnosis and mutation tracking of SARS-CoV-2 remain essential. Our strategy serves as a complete workflow of automated RNA extraction, diagnosis, and genomic surveillance that can be deployed by any laboratories with basic equipment for the current and for future viral pandemics.

## Limitations of the study

Although the throughput of the robotic MAVRICS workflow is projected to 2,000 samples daily, we have not conducted a trial to demonstrate this. The NIRVANA workflow has not been validated in clinical laboratories or received regulatory approval. One of the main reasons for this is that the ONT platform is currently restricted to research purposes only. The clinical samples used in the validation studies are all from Saudi Arabia and collected before 2023. The multiplex RPA reactions integral to NIRVANA hinge on the proficient amplification of viral targets. As the virus evolves, our current primer set may warrant revisions to maintain efficacy against emerging SARS-CoV-2 variants. While multiplex NIRVANA yields critical mutation insights, it currently exhibits lower sensitivity for SARS-CoV-2 detection compared to qRT-PCR. Efforts to enhance amplification efficiency are anticipated to achieve more comprehensive coverage for all targeted amplicons. Our workflow entails multiplex Nanopore sequencing. Special care has been taken to minimize sample crosstalk. This involves using stringent demultiplexing parameters and including an in silico NTC during demultiplexing to monitor barcode misclassification. Although no read aligned to in silico NTCs in this study, these optimizations^14^ are advisable in future applications. Basecalling error of ONT sequencing remains higher than Illumina short-read sequencing, and could cause residual sample misclassification. The ongoing development in sequencing chemistry and basecalling algorithm by Oxford Nanopore Inc. can further guarantee accurate sample demultiplexing.

## Methods

### MAVRICS clinical validation

Patient respiratory swabs (n = 94) collected in viral transport medium (VTM) were tested at King Faisal Specialist Hospital and Research Centre. The samples were plated in a 96 deep well round, and RNA was manually extracted using the MagMAX™ kit following the manufacturer’s instructions (ThermoFisher Scientific A48383) and two versions (TRIzol and Bis–Tris versions, Supplementary Material 1B, and 1C) of the MAVRICS protocol^33^ (Supplementary Material 1A). Briefly, BIS–Tris buffer (Bis–Tris version) or TRIzol and BIS–Tris buffer (TRIzol version) were added to the samples. Then, SiMNP were added and mixed by 5 min. The plates were settled on a magnetic stand and the supernatant was removed. For TRIzol version, TRIzol and BIS–Tris buffer were added, mixed, and removed. For both protocols, the SiMNP were washed four times with ethanol 90%, and dried on a microplate heater at 60 °C. Finally, nuclease-free water (40 μl) was added to the dried SiMNP, and the supernatant containing the eluted RNA was transfer a new tube. RNA from the three protocols was used to perform one-step qRT-PCR on an ABI 7500 Fast Real-Time PCR system (Applied Biosystems) with the TaqPath™ COVID‐19 CE‐IVD RT‐PCR Kit (ThermoFisher Scientific A48067) following the supplier’s instructions. To standardize the analysis of the RT-qPCR data, baseline start and end were set to 3 and 15, respectively, and in a few instances further adjusted to obtain flat background ΔRn plot. Thresholds were set at the middle of the exponential amplification phase for each target per plate. Ct values for a target were interpreted as positive if ≤ 37, and a sample considered positive if at least 2 out of 3 targets were positive. If only 1 out of 3 targets was positive the result was considered inconclusive.

### Contrived, saliva and clinical samples

Contrived samples (samples to which synthetic viral RNA was added) were prepared by mixing 1000 μl of TRIzol per each 100 μl of H_2_O, and different concentrations of in vitro transcribed SARS-CoV-2 RNA (nt28,287–29,230 in NC_045512.2l) were added. Saliva samples were prepared by mixing 1000 μl of TRIzol and 100 μl of saliva from a SARS-CoV-2 negative volunteer. Clinical samples were obtained from Ministry of Health (MOH) hospitals in the western region in Saudi Arabia. This study was approved by the Saudi Arabia Ministry of Health institutional review board (IRB# H-02-K-076-0320-279) and KAUST Institutional Biosafety and Bioethics Committee IBEC (IBEC# 20IBEC25 and 20IBEC18). All methods were carried out in accordance with relevant guidelines and regulations. Informed consent was obtained from all subjects and/or their legal guardian(s). Oropharyngeal and nasopharyngeal swabs were carried out by physicians and samples were steeped in 1 mL of TRIzol (Invitrogen Cat. No 15596018) to inactivate virus during transportation.

### Robotic RNA extraction

Robotic extraction was implemented on a Tecan EVO-200 system keeping all the steps, buffers, and RNA elution volumes similar to manual protocol (Supplementary Material 1A & 1D). The robot configuration and script are described in https://github.com/strubelab/roboRNA.

### RT-qPCR

One step R3T qRT-PCR was performed with the 2019-nCoV CDC EUA assay (nCoV_N1, nCoV_N2 and RNase P, (IDT Cat. No. 10006770)). The reaction was composed of 5 μL of 2X R3T qRT-PCR reaction buffer mix, 0.5 μL of R3T qRT-PCR enzyme mix, 0.5 μL of each probe/primer mix (nCoV_N1, nCoV_N2 or rNase P), 4 μL of eluted RNA. The reaction was performed in a CFX384 Touch Real-Time PCR System (BIO-RAD) using the following program: 55 °C 30 min, 94 °C 2 min, 45 cycles of 94 °C 15 s, 58 °C 30 s, and 68 °C 1 min; 68 °C 5 min. TaqPath™ 1-Step RT-qPCR Master Mix (ThermoFisher Scientific, A15299) was used following the supplier’s instructions.

### Library preparation and sequencing

The RPA reactions were carried out as previously described^9,10^ and purified using Agencourt AMPure XP beads (Cat No. A63881), eluted in 30 μl H_2_O, and then quantified by using Qubit. The RPA libraries were prepared using Native barcoding expansion kits (Oxford Nanopore Technologies EXP-NBD196 and SQK-NBD112-24) following Nanopore PCR tiling of SARS-CoV-2 virus with Native Barcoding protocol (Ver: PTCN_9103_v109_revN_13Jul2020). Three sequencing runs were conducted on Oxford Nanopore MinION sequencers, employing an R9.4.1 flow cell for two libraries prepared with EXP-NBD196, and an R10.4.1 flow cell for one library prepared using SQK-NBD112-24. Each run incorporated NTCs for quality control purpose.

### Bioinformatics and variant tracking

The fastq files obtained during sequencing were processed in real-time using RTNano (https://github.com/milesjor/RTNano). RTNano includes an additional round of demultiplexing using stringent parameters to reduce the chance of barcode misclassification^14^. An in silico NTC was included by RTNano to monitor barcode misclassification. The reads assigned to the in silico NTC would likely represent background errors in demultiplexing. We used the options ‘–rt_variant –align_identity = 0.95’ to ensure an alignment identity threshold of >  = 95% for reliable demultiplexing and ‘–ntc barcode99’ to ensure effective barcode misclassification monitoring^14^. For the characterization of SARS-CoV-2 variants, we referenced defining mutations as outlined on Covariants.org as of Nov 13, 2022^13^. Covariants.org serves as a comprehensive repository for SARS-CoV-2 mutation tracking, offering insight into the epidemiology and clinical impact of variant emergence and spread. It details mutation frequency by region and classifies variants according to their potential significance and impact on the virus's behavior, such as changes in transmissibility or immune evasion. These curated datasets allowed us to align our surveillance efforts with the most current understanding of variant dynamics at the time of analysis. Additional processing details and the utility of RTNano are described in our previous publications^10,14^.

### SARS-CoV-2 genome sequencing and genome assembly

Samples were prepared for sequencing using the SARS-CoV-2 genome sequencing protocol midnight with Oxford Nanopore Rapid barcoding kit (https://www.protocols.io/view/sars-cov2-genome-sequencing-protocol-1200bp-amplic-rm7vz8q64vx1/v6). The artic primer set V4.1 (https://github.com/joshquick/artic-ncov2019/tree/master/primer_schemes/nCoV-2019/V3) was used to generate 400 bp amplicon instead of using midnight 1200 bp amplicon. The Oxford Nanopore Rapid Barcoding kit (https://store.nanoporetech.com/productDetail/?id=rapid-barcoding-kit-1) were used for barcoding and multiplexing samples. The barcoded samples were then loaded and sequenced on MinION MK1C platform. Generated data were analyzed using the wf-artic pipeline from Epi2me-labs (https://github.com/epi2me-labs/wf-artic) and the SARS-CoV-2 variants were called.

### Supplementary Information

Supplementary Information.

## Data Availability

All data analyzed during this study are included in this published article and its supplementary information files. Raw sequencing datasets used and/or analyzed during the current study are available from the SRA database (accession ID PRJNA1031940) via the following link: https://www.ncbi.nlm.nih.gov/bioproject/PRJNA1031940.
