# Supplementary material for: An open-source, automated, and cost-effective platform for COVID-19 diagnosis and rapid portable genomic surveillance using nanopore sequencing
Source: Sci Rep. 2023 Nov 21;13:20349. doi: 10.1038/s41598-023-47190-w (PMC10663496; doi:10.1038/s41598-023-47190-w)

**SUPPLEMENTARY FIGURES**

**SUPPLEMENTARY FIGURE LEGEND**

**Supplementary Figure 1. Reproducibility in the synthesis of SiMNP and preparation of Bis-Tris buffer.** RNA was extracted from contrived SARS-CoV-2 RNA samples (1x10^6^ RNA copies/input sample) by MAVRICS protocol using different batches of SiMNP (A) or Bis-Tris buffer (B). The extracted RNA was used in the RT-qPCR reaction using TaqPath™ 1-Step RT-qPCR and the probe nCoV_N1.

**Supplementary Figure 2. R3T qRT-PCR system validation and performance.** The absolute limit of detection (LoD) was evaluated for R3T qRT-PCR system in parallel with the commercial TaqPath™. Synthetic SAR-CoV-2 RNA (1, 5, 50 and 100 RNA copies) were used as input in the RT-qPCR reactions and the probes nCoV_N1 and nCoV_N2 detected by both systems. Ct values for R3T qRT-PCR system A) and TaqPath™(B). C. Number of replicates detected/total of replicates and percentages of detection for nCoV_N1 and nCoV_N2. The absolute LoD (RNA copies/reaction) was determined considering the point where the signal is detected in ≥95% of the samples with a Ct value lower than 40.

**Supplementary Figure 3**. **Primer sequences and verification of RPA amplicons specificity using restriction enzymes.** A. Primer sequences for RPA assays targeting SARS-CoV-2 spike gene mutational hotspots and for the internal control ACTB. B**.** cDNA was generated by reverse transcription from one positive patient sample, singleplex RPA assay were done for each primer pair, and enzymatic reactions were conducted in the absence or the presence of specific restriction enzymes. Yellow arrows indicate 300 bp in the DNA ladder and asterisks indicate DNA of expected sizes after treatment with each restriction enzyme. Original gels are presented in Supplementary Material 7.

**SUPPLEMENTARY MATERIALS**

**Supplementary Material 1. Magnetic nanoparticle-Aided Viral RNA Isolation of Contagious Samples (MAVRICS) protocol original and adapted versions.** MAVRICS original protocol (A), MAVRICS-TRIzol version (B), MAVRICS Bis-Tris version (C), and Robotic protocol for RNA extraction by MAVRCIS (D). Reagents, SiMNPs and buffers preparation are available in protocol.io^32^.

**Supplementary Material 1A. MAVRICS original protocol.**

**A. Reagents**

1. Bis-Tris buffer:

- 10 mM Bis-Tris /HCl pH < 6.5, 3M guanidium hydrochloride, in 90% Ethanol

2. SiMNP diluted (1:4 with RNase-free water) in 96-well plate

3. 90% Ethanol

**B. Steps**

Oropharyngeal or nasopharyngeal swabs are steeped in acid guanidinium thiocyanate-phenol-chloroform (AGPC, e.g., TRIzol Reagent or TRI reagent).

- In an Eppendorf tube, add 200 µl clinical sample steeped in AGPC (TRIzol or TRI reagent) and 200 µl Bis-Tris buffer, mix well by vortexing.
- Add 40 µl of diluted (1:4 with RNase-free water) SiMNP, mix 5 min at 1300 rpm.
- Spin the tube for 2-3 seconds, settle the SiMNPs on a magnetic stand and remove the supernatant. Remove the tube from the magnetic stand
- Mix 200 µl of AGPC (TRIzol or TRI reagent) and 200 µl Bis-Tris buffer, add to the SiMNPs, mix well by vortexing.
- Settle the SiMNPs on a magnetic stand and remove the supernatant. Remove the tube from the magnetic stand.
- Add 400 µl of 90% ethanol, spin for 2-3 seconds, settle the SiMNPs on a magnetic stand and remove the supernatant. Remove the tube from the magnetic stand.
- Repeat Step 6 three more times for a total of 4 ethanol washes.
- After removing the supernatant from the last ethanol wash, dry the SiMNPs on a heat block at 50°C. Keep the lid open, no shaking. Do not elute before the SiMNPs are dried.
- To elute the RNA, add 40 µl nuclease-free water, and mix 5 min at 1300 rpm at room temperature.
- Settle the SiMNPs on a magnetic stand and transfer the eluted RNA to a new RNase-free tube.
- Store RNA at -80°C or use immediately.

**Supplementary Material 1B. MAVRICS-TRIzol version**

**A. Reagents**

1. Bis-Tris buffer:

- 10 mM Bis-Tris /HCl pH < 6.5, 3M guanidium hydrochloride, in 90% Ethanol

2. SiMNP diluted (1:4 with RNase-free water) in 96-well plate

3. 90% Ethanol

**B. Steps**

Respiratory samples steeped in virus transport medium (VTM).

- In a 96 deep well round bottom plate add 100 µl of sample steeped in VTM and 300 µl of TRizol, mix well
- Add 400 µl Bis-Tris buffer, and mix well at 1300 rpm in a thermomixer or a microplate shaker for 20 seconds.
- Add 40 µl of diluted (1:4 with RNase-free water) SiMNPs, mix at 1300 rpm in a thermomixer or a microplate shaker for 5 min.
- Settle the samples on a magnetic stand for 10 minutes or until all of the beads have been collected and remove and discard the supernatant. Avoid disturbing the SiMNP pellet.
- For each well, mix 200 µl of TRIzol and 200 µl Bis-Tris buffer, and add to the SiMNPs (400 µl), and mix well at 1300 rpm in a thermomixer or a microplate shaker for 20 seconds.
- Settle the samples on a magnetic stand for 3 minutes or until all of the beads have been collected and remove the supernatant. Avoid disturbing the SiMNP pellet.
- Add 400 µl of 90% ethanol, settle samples on a magnetic stand for 2 minutes or until all of the beads have collected, and remove and discard the supernatant. Avoid disturbing the SiMNP pellet.
  - [It is highly recommended to prepare fresh 90% ethanol before use. Make sure the 90% ethanol container is closed tightly to prevent evaporation.]
- Repeat Step 7 three more times for a total of 4 ethanol washes.
- After discarding the supernatant from the last ethanol wash, dry the SiMNPs on a microplate heater at 60°C (20 min). Keep the lid open, no shaking. Do not elute before the SiMNPs are dried.
  - [Drying may take less than 20 min. Monitor the appearance of the SiMNPs during drying. The SiMNPs appear in a rusty brownish color when dried.]
- To elute the RNA, add 40 µl nuclease-free water, and mix at 1300 rpm in a thermomixer or a microplate shaker for 5 min at room temperature.
- Settle the tube on a magnetic stand for 3 minutes or until all of the beads have been collected and transfer the eluted RNA (~30 µl) to a new RNase-free tube. Avoid disturbing the SiMNP pellet.
- Store RNA at -80°C or use immediately.

**Supplementary Material 1C. MAVRICS BisTris version**

**A. Reagents**

1. Bis-Tris buffer:

- 10 mM Bis-Tris /HCl pH < 6.5, 3M guanidium hydrochloride, in 90% Ethanol

2. SiMNP diluted (1:4 with RNase-free water) in 96-well plate

3. 90% Ethanol

**B. Steps**

Respiratory samples steeped in virus transport medium (VTM).

- In a 96 deep well round bottom plate add 200 µl of sample steeped in VTM and 400 µl of Bis-Tris buffer, mix well at 1300 rpm in a thermomixer or a microplate shaker for 20 seconds.
- Add 40 µl of diluted (1:4 with RNase-free water) SiMNPs, mix at 1300 rpm in a thermomixer for 5 min.
- Settle the samples on a magnetic stand for 10 minutes or until all of the beads have been collected and remove and discard the supernatant. Avoid disturbing the SiMNP pellet.
- Add 400 µl of 90% ethanol to the sample, mix at 1300 rpm in a thermomixer or a microplate shaker for 20 seconds, settle the tube on a magnetic stand for 2 minutes or until all of the beads have collected, and remove and discard the supernatant. Avoid disturbing the SiMNP pellet.
- [It is highly recommended to prepare fresh 90% ethanol before use. Make sure the 90% ethanol container is closed tightly to prevent evaporation.]
- Repeat Step 4 three more times for a total of 4 ethanol washes.
- After discarding the supernatant from the last ethanol wash, dry the SiMNPs on a microplate heater at 60°C (20 min). Keep the lid open, no shaking. Do not elute before the SiMNPs are dried.
- [Drying may take less than 20 min. Monitor the appearance of the SiMNPs during drying. The SiMNPs appear in a rusty brownish color when dried.]

- To elute the RNA, add 40 µl nuclease-free water, and mix at 1300 rpm in a thermomixer or a microplate shaker for 5 min at room temperature.
- Settle the tube on a magnetic stand for 3 minutes or until all of the beads have collected, and transfer the eluted RNA (~30 µl) to a new RNase-free tube. Avoid disturbing the SiMNP pellet.
- Store RNA at -80°C or use immediately.

**Supplementary Material 1D. Robotic protocol for RNA extraction by MAVRCIS**

**Reagents**

1. Binding buffer (Bis-Tris buffer):

- 10 mM Bis-Tris /HCl pH< 6.5, 3M guanidium hydrochloride, in 90% Ethanol

2. SiMNP diluted (1:4 with RNase-free water) in 96-well plate (60µl/well)

3. Wash buffer:

- Binding buffer + TRIzol at 1:1 v/v

4. Ethanol wash

- 90% Ethanol

**B. Materials**

1. Magnet for bead separation in plates (V&P Scientific, Inc. Cat. VP 771MDWZM-1-ALT)

2. Patient Sample plate (96 deepwell). Any deepwell plate. Same plates can be used for 2 waste positions

3. Extraction-plate (96 deepwell, square-well, round-bottom) (Whatman, Cat. 7701-5200)

4. 96-well plate for beads input (60µl of SiMNP/well, regular 96-well, round-bottom) e.g. Corning Cat. 3788

5. Universal microplate lids (Whatman / Cytivia Cat. 77041001)

6’ Evo MCA96 nested tips 200µl sterile (Axygen, Cat. EV-200-NTR-S)

**C. TECAN EVO Script Summary.** All Evoware scripts, configuration, and workflow are published at: <https://github.com/strubelab/roboRNA>

- Volumes are per well
- All plates (except 2^nd^ waste position) are covered with lids, to be removed before each pipetting step and placed back afterwards

**Phase 1. RNA Binding**

- Pickup MCA tips
- Add 300µl (2x150µl) binding buffer to extraction plate
- Briefly shake 96-well plate containing beads (resuspension)
- Transfer 40µl beads solution to extraction plate (pipette up / down on aspiration)
- Transfer 150µl (2x) patient sample prepared in TRIzol reagent from input plate to Extraction plate, shake 5 min @ 1300 rpm
- Move extraction plate to magnet, 30s – 1 min settling time
- Aspirate and discard supernatant
- Move extraction plate back to regular pipetting position
- Change MCA tips

**Wash I**

- Add 150µl (2x) wash buffer, shake 20s @ 1300 rpm
- Move extraction plate to magnet, 30s – 1 min settling time
- Aspirate and discard supernatant
- Move extraction plate back to regular pipetting position
- Change MCA tips.

**Ethanol wash repeat 4x**

- Add 150µl (2x) 90% EtOH, shake 20s @ 1300 rpm
- Move extraction plate to magnet, 30s – 1 min settling time
- Aspirate and discard supernatant
- Move extraction plate back to regular pipetting position
- Change MCA tips (optional between steps)

**Drying**

- Move extraction plate to heat block, incubate 20 min @ 50 C:
- Move extraction plate back to regular pipetting position

**Phase 5. Elution**

- Add 40µl nuclease-free water to extraction plate wells
- Shake 5 min @ 1300 rpm
- Move extraction plate to magnet, 30s – 1 min settling time
- Move destination plate from hotel to pipetting position, remove lid
- Transfer supernatant from extraction plate to destination plate, place back lid
- Discard tips

Store RNA at -80°C or use immediately.

**Supplementary Material 2.** **MAVRICS protocol validation Ct values and diagnosis**

RNA was extracted in parallel by MAVRICS protocol MAVRICS Bis-Tris version, TRIzol version and MagMAX™ Kit (Thermo Fisher Scientific A48383) from 94 clinical samples. COVID-19 was detected by TaqPath™ COVID-19 Combo Kit (N gene, S gene, ORF 1ab, and the internal control MS2). The Ct values were obtained from the same threshold for the protocols. The criterion for viral target detection and COVID-19 diagnosis are described in Methods section. UD (undetected).

|  |  | **MagMax** |  | **TRIzol** |  | **BisTris** |  |
| --- | --- | --- | --- | --- | --- | --- | --- |
| **Sample Name** | **Target Name** | **Cт** |  | **Cт** |  | **Cт** |  |
| Sample 1 | MS2 | 26.0 |  | 26.7 |  | 25.3 |  |
| Sample 1 | N gene | 34.2 |  | 36.7 |  | 36.7 |  |
| Sample 1 | ORF1ab | 34.7 |  | 35.7 |  | UD |  |
| Sample 1 | S gene | 34.5 | POS | UD | POS | 33.7 | POS |
| Sample 2 | MS2 | 26.3 |  | 26.8 |  | 25.4 |  |
| Sample 2 | N gene | 22.0 |  | 23.9 |  | 22.4 |  |
| Sample 2 | ORF1ab | 21.9 |  | 23.7 |  | 22.0 |  |
| Sample 2 | S gene | 22.8 | POS | 25.3 | POS | 21.7 | POS |
| Sample 3 | MS2 | 26.2 |  | 26.6 |  | 25.0 |  |
| Sample 3 | N gene | 22.6 |  | 25.0 |  | 24.2 |  |
| Sample 3 | ORF1ab | 23.3 |  | 25.5 |  | 24.7 |  |
| Sample 3 | S gene | 23.0 | POS | 26.1 | POS | 23.4 | POS |
| Sample 4 | MS2 | 25.6 |  | 26.6 |  | 25.2 |  |
| Sample 4 | N gene | 26.5 |  | 27.7 |  | 26.4 |  |
| Sample 4 | ORF1ab | 26.9 |  | 28.3 |  | 26.9 |  |
| Sample 4 | S gene | 26.5 | POS | 28.8 | POS | 25.5 | POS |
| Sample 5 | MS2 | 25.6 |  | 26.5 |  | 24.8 |  |
| Sample 5 | N gene | 22.7 |  | 24.6 |  | 23.9 |  |
| Sample 5 | ORF1ab | 22.4 |  | 23.9 |  | 24.1 |  |
| Sample 5 | S gene | 22.4 | POS | 25.7 | POS | 23.2 | POS |
| Sample 6 | MS2 | 25.6 |  | 26.5 |  | 24.7 |  |
| Sample 6 | N gene | 26.8 |  | 27.0 |  | 25.4 |  |
| Sample 6 | ORF1ab | 27.3 |  | 27.5 |  | 25.8 |  |
| Sample 6 | S gene | 27.0 | POS | 27.9 | POS | 24.4 | POS |
| Sample 7 | MS2 | 25.4 |  | 26.2 |  | 24.1 |  |
| Sample 7 | N gene | 24.5 |  | 28.3 |  | 27.0 |  |
| Sample 7 | ORF1ab | 24.3 |  | 28.8 |  | 27.1 |  |
| Sample 7 | S gene | 25.0 | POS | 29.8 | POS | 26.7 | POS |
| Sample 8 | MS2 | 25.8 |  | 27.0 |  | 25.2 |  |
| Sample 8 | N gene | 35.5 |  | UD |  | 36.3 |  |
| Sample 8 | ORF1ab | 35.5 |  | UD |  | 38.9 |  |
| Sample 8 | S gene | 36.0 | POS | UD | NEG | 36.5 | POS |
| Sample 9 | MS2 | 26.1 |  | 27.2 |  | 25.0 |  |
| Sample 9 | N gene | 21.4 |  | 23.1 |  | 22.9 |  |
| Sample 9 | ORF1ab | 24.5 |  | 25.8 |  | 26.3 |  |
| Sample 9 | S gene | 25.2 | POS | 27.4 | POS | 26.0 | POS |
| Sample 10 | MS2 | 25.8 |  | 27.2 |  | 25.7 |  |
| Sample 10 | N gene | 33.7 |  | 35.6 |  | 34.7 |  |
| Sample 10 | ORF1ab | 33.6 |  | 34.4 |  | 35.5 |  |
| Sample 10 | S gene | 34.1 | POS | 37.7 | POS | 33.7 | POS |
| Sample 11 | MS2 | 26.0 |  | 29.8 |  | 25.2 |  |
| Sample 11 | N gene | 30.6 |  | 33.7 |  | 31.8 |  |
| Sample 11 | ORF1ab | 31.7 |  | 34.2 |  | 32.2 |  |
| Sample 11 | S gene | 32.0 | POS | 38.1 | POS | 31.0 | POS |
| Sample 12 | MS2 | 25.5 |  | 26.9 |  | 25.5 |  |
| Sample 12 | N gene | 23.5 |  | 35.5 |  | 35.7 |  |
| Sample 12 | ORF1ab | 23.8 |  | 36.6 |  | 37.9 |  |
| Sample 12 | S gene | 23.8 | POS | UD | POS | 34.5 | POS |
| Sample 13 | MS2 | 28.1 |  | 27.5 |  | 25.6 |  |
| Sample 13 | N gene | 18.7 |  | 21.3 |  | 20.1 |  |
| Sample 13 | ORF1ab | 18.8 |  | 21.4 |  | 20.9 |  |
| Sample 13 | S gene | 18.6 | POS | 22.2 | POS | 19.8 | POS |
| Sample 14 | MS2 | 26.4 |  | 27.0 |  | 25.3 |  |
| Sample 14 | N gene | 22.0 |  | 24.3 |  | 24.3 |  |
| Sample 14 | ORF1ab | 21.9 |  | 24.2 |  | 24.6 |  |
| Sample 14 | S gene | 21.6 | POS | 24.9 | POS | 23.3 | POS |
| Sample 15 | MS2 | 26.3 |  | 27.0 |  | 25.5 |  |
| Sample 15 | N gene | 24.2 |  | 26.1 |  | 25.4 |  |
| Sample 15 | ORF1ab | 24.9 |  | 26.5 |  | 26.0 |  |
| Sample 15 | S gene | 24.5 | POS | 27.3 | POS | 24.7 | POS |
| Sample 16 | MS2 | 29.2 |  | 28.8 |  | 26.1 |  |
| Sample 16 | N gene | 18.4 |  | 19.7 |  | 19.7 |  |
| Sample 16 | ORF1ab | 18.7 |  | 19.9 |  | 20.1 |  |
| Sample 16 | S gene | 19.5 | POS | 21.5 | POS | 19.9 | POS |
| Sample 17 | MS2 | 26.0 |  | 27.1 |  | 25.3 |  |
| Sample 17 | N gene | 31.4 |  | 33.0 |  | 32.0 |  |
| Sample 17 | ORF1ab | 31.6 |  | 33.0 |  | 32.2 |  |
| Sample 17 | S gene | 31.0 | POS | 33.6 | POS | 30.8 | POS |
| Sample 18 | MS2 | 27.4 |  | 27.3 |  | 25.2 |  |
| Sample 18 | N gene | 19.7 |  | 22.7 |  | 21.9 |  |
| Sample 18 | ORF1ab | 19.5 |  | 22.8 |  | 21.9 |  |
| Sample 18 | S gene | 19.1 | POS | 23.1 | POS | 20.4 | POS |
| Sample 19 | MS2 | 25.9 |  | 27.4 |  | 25.5 |  |
| Sample 19 | N gene | 33.4 |  | 35.8 |  | 39.7 |  |
| Sample 19 | ORF1ab | 33.2 |  | 36.1 |  | 35.6 |  |
| Sample 19 | S gene | 33.8 | POS | UD | POS | 33.0 | POS |
| Sample 20 | MS2 | 25.8 |  | 26.9 |  | 25.4 |  |
| Sample 20 | N gene | 23.3 |  | 22.1 |  | 25.8 |  |
| Sample 20 | ORF1ab | 24.0 |  | 21.9 |  | 27.6 |  |
| Sample 20 | S gene | 23.6 | POS | 22.2 | POS | 26.2 | POS |
| Sample 21 | MS2 | 26.3 |  | 28.1 |  | 25.5 |  |
| Sample 21 | N gene | 23.8 |  | 26.2 |  | 26.2 |  |
| Sample 21 | ORF1ab | 24.3 |  | 26.6 |  | 26.8 |  |
| Sample 21 | S gene | 23.8 | POS | 26.9 | POS | 25.2 | POS |
| Sample 22 | MS2 | 26.2 |  | 28.1 |  | 25.6 |  |
| Sample 22 | N gene | 31.5 |  | 33.8 |  | 32.2 |  |
| Sample 22 | ORF1ab | 32.1 |  | 33.6 |  | 34.2 |  |
| Sample 22 | S gene | 31.8 | POS | 31.3 | POS | 32.0 | POS |
| Sample 23 | MS2 | 26.1 |  | 26.9 |  | 25.5 |  |
| Sample 23 | N gene | 31.1 |  | 29.1 |  | 31.3 |  |
| Sample 23 | ORF1ab | 31.7 |  | 28.6 |  | 31.6 |  |
| Sample 23 | S gene | 31.4 | POS | 28.8 | POS | 30.0 | POS |
| Sample 24 | MS2 | 27.8 |  | 27.6 |  | 25.7 |  |
| Sample 24 | N gene | 18.6 |  | 20.4 |  | 19.9 |  |
| Sample 24 | ORF1ab | 19.0 |  | 20.7 |  | 21.2 |  |
| Sample 24 | S gene | 18.9 | POS | 21.5 | POS | 20.3 | POS |
| Sample 25 | MS2 | 26.7 |  | 27.2 |  | 25.4 |  |
| Sample 25 | N gene | 21.5 |  | 23.4 |  | 24.0 |  |
| Sample 25 | ORF1ab | 21.5 |  | 23.1 |  | 24.2 |  |
| Sample 25 | S gene | 21.3 | POS | 23.7 | POS | 22.9 | POS |
| Sample 26 | MS2 | 26.3 |  | 27.2 |  | 25.6 |  |
| Sample 26 | N gene | 30.3 |  | 31.6 |  | 34.0 |  |
| Sample 26 | ORF1ab | 30.4 |  | 31.6 |  | 34.2 |  |
| Sample 26 | S gene | 30.0 | POS | 32.3 | POS | 32.9 | POS |
| Sample 27 | MS2 | 26.4 |  | 27.2 |  | 25.7 |  |
| Sample 27 | N gene | 25.8 |  | 24.8 |  | 26.9 |  |
| Sample 27 | ORF1ab | 26.1 |  | 24.6 |  | 26.6 |  |
| Sample 27 | S gene | 25.7 | POS | 25.1 | POS | 24.9 | POS |
| Sample 28 | MS2 | 29.1 |  | 28.1 |  | 26.9 |  |
| Sample 28 | N gene | 18.5 |  | 19.9 |  | 19.7 |  |
| Sample 28 | ORF1ab | 19.2 |  | 20.1 |  | 20.1 |  |
| Sample 28 | S gene | 18.8 | POS | 20.6 | POS | 18.7 | POS |
| Sample 29 | MS2 | 26.2 |  | 27.2 |  | 25.3 |  |
| Sample 29 | N gene | 29.4 |  | 30.7 |  | 32.2 |  |
| Sample 29 | ORF1ab | 29.8 |  | 31.3 |  | 33.0 |  |
| Sample 29 | S gene | 29.5 | POS | 31.5 | POS | 31.6 | POS |
| Sample 30 | MS2 | 26.2 |  | 27.4 |  | 25.2 |  |
| Sample 30 | N gene | 25.5 |  | 26.0 |  | 27.5 |  |
| Sample 30 | ORF1ab | 25.6 |  | 26.0 |  | 27.2 |  |
| Sample 30 | S gene | 26.3 | POS | 27.3 | POS | 26.7 | POS |
| Sample 31 | MS2 | 26.0 |  | 27.8 |  | 25.6 |  |
| Sample 31 | N gene | 24.1 |  | 24.6 |  | 25.6 |  |
| Sample 31 | ORF1ab | 24.4 |  | 24.8 |  | 26.0 |  |
| Sample 31 | S gene | 24.1 | POS | 25.4 | POS | 24.7 | POS |
| Sample 32 | MS2 | 25.9 |  | 27.9 |  | 25.3 |  |
| Sample 32 | N gene | 37.7 |  | UD |  | UD |  |
| Sample 32 | ORF1ab | UD |  | 39.2 |  | UD |  |
| Sample 32 | S gene | UD | NEG | UD | NEG | UD | NEG |
| Sample 33 | MS2 | 26.5 |  | 27.8 |  | 25.8 |  |
| Sample 33 | N gene | 26.2 |  | 26.2 |  | 26.5 |  |
| Sample 33 | ORF1ab | 26.7 |  | 25.8 |  | 26.1 |  |
| Sample 33 | S gene | 27.1 | POS | 27.0 | POS | 25.6 | POS |
| Sample 34 | MS2 | 26.3 |  | 28.4 |  | 25.8 |  |
| Sample 34 | N gene | 26.6 |  | 28.2 |  | 30.0 |  |
| Sample 34 | ORF1ab | 26.8 |  | 28.5 |  | 30.0 |  |
| Sample 34 | S gene | 27.4 | POS | 29.5 | POS | 29.2 | POS |
| Sample 35 | MS2 | 32.7 |  | 30.4 |  | 26.8 |  |
| Sample 35 | N gene | 17.1 |  | 19.2 |  | 19.9 |  |
| Sample 35 | ORF1ab | 18.3 |  | 19.8 |  | 20.9 |  |
| Sample 35 | S gene | 18.8 | POS | 21.6 | POS | 20.8 | POS |
| Sample 36 | MS2 | 26.9 |  | 27.3 |  | 25.9 |  |
| Sample 36 | N gene | 21.0 |  | 22.5 |  | 21.6 |  |
| Sample 36 | ORF1ab | 20.7 |  | 22.2 |  | 21.4 |  |
| Sample 36 | S gene | 21.5 | POS | 23.8 | POS | 21.1 | POS |
| Sample 37 | MS2 | 26.3 |  | 27.1 |  | 25.6 |  |
| Sample 37 | N gene | 38.5 |  | 35.9 |  | 37.7 |  |
| Sample 37 | ORF1ab | UD |  | 36.8 |  | 38.2 |  |
| Sample 37 | S gene | UD | NEG | UD | POS | 34.1 | REP |
| Sample 38 | MS2 | 27.2 |  | 28.0 |  | 25.7 |  |
| Sample 38 | N gene | 20.5 |  | 22.7 |  | 23.2 |  |
| Sample 38 | ORF1ab | 21.0 |  | 23.4 |  | 24.1 |  |
| Sample 38 | S gene | 21.7 | POS | 24.8 | POS | 23.7 | POS |
| Sample 39 | MS2 | 26.6 |  | 27.4 |  | 25.6 |  |
| Sample 39 | N gene | 24.0 |  | 26.8 |  | 25.8 |  |
| Sample 39 | ORF1ab | 23.7 |  | 26.6 |  | 26.1 |  |
| Sample 39 | S gene | 23.3 | POS | 27.0 | POS | 24.6 | POS |
| Sample 40 | MS2 | 31.5 |  | 29.8 |  | 26.5 |  |
| Sample 40 | N gene | 17.0 |  | 18.9 |  | 19.0 |  |
| Sample 40 | ORF1ab | 18.3 |  | 20.4 |  | 20.6 |  |
| Sample 40 | S gene | 17.8 | POS | 20.7 | POS | 19.1 | POS |
| Sample 41 | MS2 | 26.4 |  | 27.7 |  | 25.8 |  |
| Sample 41 | N gene | 23.3 |  | 22.2 |  | 23.2 |  |
| Sample 41 | ORF1ab | 23.7 |  | 22.6 |  | 23.2 |  |
| Sample 41 | S gene | 24.3 | POS | 24.0 | POS | 22.7 | POS |
| Sample 42 | MS2 | 25.9 |  | 26.8 |  | 25.3 |  |
| Sample 42 | N gene | 26.9 |  | 26.6 |  | 29.1 |  |
| Sample 42 | ORF1ab | 27.4 |  | 26.7 |  | 29.1 |  |
| Sample 42 | S gene | 26.9 | POS | 27.3 | POS | 27.5 | POS |
| Sample 43 | MS2 | 26.4 |  | 27.3 |  | 25.6 |  |
| Sample 43 | N gene | 31.1 |  | 32.4 |  | 33.4 |  |
| Sample 43 | ORF1ab | 31.9 |  | 32.1 |  | 33.2 |  |
| Sample 43 | S gene | 31.9 | POS | 32.5 | POS | 30.4 | POS |
| Sample 44 | MS2 | 26.6 |  | 28.0 |  | 25.9 |  |
| Sample 44 | N gene | 29.9 |  | 31.6 |  | 32.2 |  |
| Sample 44 | ORF1ab | 30.6 |  | 32.9 |  | 32.9 |  |
| Sample 44 | S gene | 30.6 | POS | 32.1 | POS | 31.6 | POS |
| Sample 45 | MS2 | 26.7 |  | 27.9 |  | 26.1 |  |
| Sample 45 | N gene | 28.6 |  | 29.0 |  | 27.4 |  |
| Sample 45 | ORF1ab | 28.2 |  | 28.4 |  | 27.5 |  |
| Sample 45 | S gene | 28.7 | POS | 29.0 | POS | 26.9 | POS |
| Sample 46 | MS2 | 26.9 |  | 27.5 |  | 26.4 |  |
| Sample 46 | N gene | 23.8 |  | 24.6 |  | 22.9 |  |
| Sample 46 | ORF1ab | 24.0 |  | 24.1 |  | 23.3 |  |
| Sample 46 | S gene | 24.6 | POS | 25.2 | POS | 22.9 | POS |
| Sample 47 | MS2 | 26.9 |  | 28.9 |  | 26.3 |  |
| Sample 47 | N gene | 28.3 |  | 30.1 |  | 28.3 |  |
| Sample 47 | ORF1ab | 28.5 |  | 30.4 |  | 29.0 |  |
| Sample 47 | S gene | 27.9 | POS | 30.8 | POS | 27.5 | POS |
| Sample 48 | MS2 | 26.7 |  | 27.3 |  | 25.9 |  |
| Sample 48 | N gene | 27.0 |  | 29.5 |  | 29.6 |  |
| Sample 48 | ORF1ab | 27.4 |  | 29.9 |  | 30.6 |  |
| Sample 48 | S gene | 27.2 | POS | 30.5 | POS | 29.4 | POS |
| Sample 49 | MS2 | 35.9 |  | 30.0 |  | 30.0 |  |
| Sample 49 | N gene | 15.5 |  | 21.1 |  | 16.8 |  |
| Sample 49 | ORF1ab | 16.0 |  | 21.0 |  | 17.0 |  |
| Sample 49 | S gene | 15.8 | POS | 21.4 | POS | 16.0 | POS |
| Sample 50 | MS2 | 26.8 |  | 30.4 |  | 25.7 |  |
| Sample 50 | N gene | UD |  | 38.2 |  | 30.8 |  |
| Sample 50 | ORF1ab | UD |  | UD |  | 31.3 |  |
| Sample 50 | S gene | UD | NEG | UD | NEG | 29.8 | POS |
| Sample 51 | MS2 | 27.6 |  | 27.4 |  | 25.8 |  |
| Sample 51 | N gene | 22.6 |  | 24.9 |  | 23.1 |  |
| Sample 51 | ORF1ab | 22.8 |  | 24.7 |  | 23.1 |  |
| Sample 51 | S gene | 22.3 | POS | 25.2 | POS | 21.8 | POS |
| Sample 52 | MS2 | 26.7 |  | 27.3 |  | 25.7 |  |
| Sample 52 | N gene | 22.9 |  | 25.1 |  | 23.9 |  |
| Sample 52 | ORF1ab | 22.9 |  | 24.8 |  | 24.3 |  |
| Sample 52 | S gene | 22.5 | POS | 25.0 | POS | 22.7 | POS |
| Sample 53 | MS2 | 26.4 |  | 27.2 |  | 25.3 |  |
| Sample 53 | N gene | 30.1 |  | 32.1 |  | 31.9 |  |
| Sample 53 | ORF1ab | 31.0 |  | 32.7 |  | 33.4 |  |
| Sample 53 | S gene | 30.4 | POS | 33.4 | POS | 31.5 | POS |
| Sample 54 | MS2 | 26.0 |  | 26.9 |  | 25.1 |  |
| Sample 54 | N gene | 35.3 |  | UD |  | 32.5 |  |
| Sample 54 | ORF1ab | UD |  | 39.5 |  | 33.0 |  |
| Sample 54 | S gene | UD | REP | 37.2 | NEG | 31.1 | POS |
| Sample 55 | MS2 | 26.4 |  | 27.2 |  | 25.4 |  |
| Sample 55 | N gene | 23.6 |  | 24.6 |  | 24.3 |  |
| Sample 55 | ORF1ab | 25.4 |  | 26.0 |  | 26.6 |  |
| Sample 55 | S gene | 24.9 | POS | 26.4 | POS | 25.3 | POS |
| Sample 56 | MS2 | 26.5 |  | 27.3 |  | 25.9 |  |
| Sample 56 | N gene | 23.1 |  | 23.0 |  | 22.6 |  |
| Sample 56 | ORF1ab | 24.0 |  | 23.2 |  | 23.5 |  |
| Sample 56 | S gene | UD | POS | 33.0 | POS | UD | POS |
| Sample 57 | MS2 | 26.7 |  | 27.5 |  | 26.3 |  |
| Sample 57 | N gene | 21.6 |  | 22.9 |  | 20.5 |  |
| Sample 57 | ORF1ab | 22.3 |  | 22.8 |  | 21.4 |  |
| Sample 57 | S gene | 21.9 | POS | 23.2 | POS | 20.2 | POS |
| Sample 58 | MS2 | 26.9 |  | 28.2 |  | 26.1 |  |
| Sample 58 | N gene | 25.4 |  | 28.0 |  | 26.7 |  |
| Sample 58 | ORF1ab | 25.6 |  | 27.9 |  | 27.2 |  |
| Sample 58 | S gene | 25.2 | POS | 28.2 | POS | 25.7 | POS |
| Sample 59 | MS2 | 26.9 |  | 27.7 |  | 26.2 |  |
| Sample 59 | N gene | 31.3 |  | 30.8 |  | 31.8 |  |
| Sample 59 | ORF1ab | 31.8 |  | 30.7 |  | 32.5 |  |
| Sample 59 | S gene | 31.2 | POS | 31.1 | POS | 30.9 | POS |
| Sample 60 | MS2 | 26.9 |  | 27.5 |  | 26.2 |  |
| Sample 60 | N gene | 28.0 |  | 28.7 |  | 28.3 |  |
| Sample 60 | ORF1ab | 28.3 |  | 28.9 |  | 28.7 |  |
| Sample 60 | S gene | 28.0 | POS | 29.2 | POS | 27.0 | POS |
| Sample 61 | MS2 | 26.5 |  | 27.3 |  | 25.5 |  |
| Sample 61 | N gene | 32.9 |  | 34.4 |  | 29.8 |  |
| Sample 61 | ORF1ab | 33.3 |  | 36.2 |  | 29.4 |  |
| Sample 61 | S gene | 33.8 | POS | UD | POS | 27.7 | POS |
| Sample 62 | MS2 | 26.5 |  | 27.4 |  | 25.8 |  |
| Sample 62 | N gene | 31.7 |  | 31.1 |  | 30.5 |  |
| Sample 62 | ORF1ab | 32.0 |  | 31.1 |  | 30.3 |  |
| Sample 62 | S gene | 31.7 | POS | 31.5 | POS | 28.5 | POS |
| Sample 63 | MS2 | 26.8 |  | 27.4 |  | 25.8 |  |
| Sample 63 | N gene | UD |  | 36.3 |  | 33.8 |  |
| Sample 63 | ORF1ab | UD |  | 37.8 |  | 34.1 |  |
| Sample 63 | S gene | UD | NEG | 34.1 | POS | 32.7 | POS |
| Sample 64 | MS2 | 30.8 |  | 32.4 |  | 25.7 |  |
| Sample 64 | N gene | 18.0 |  | 18.3 |  | 18.5 |  |
| Sample 64 | ORF1ab | 18.6 |  | 18.3 |  | 18.9 |  |
| Sample 64 | S gene | 18.2 | POS | 18.7 | POS | 17.7 | POS |
| Sample 65 | MS2 | 26.0 |  | 26.5 |  | 24.9 |  |
| Sample 65 | N gene | 39.4 |  | 33.1 |  | 34.7 |  |
| Sample 65 | ORF1ab | UD |  | 33.0 |  | 35.4 |  |
| Sample 65 | S gene | UD | NEG | 34.0 | POS | 33.9 | POS |
| Sample 66 | MS2 | 25.9 |  | 26.8 |  | 25.0 |  |
| Sample 66 | N gene | 30.4 |  | 34.9 |  | 34.9 |  |
| Sample 66 | ORF1ab | 31.1 |  | 35.3 |  | 36.0 |  |
| Sample 66 | S gene | 30.8 | POS | 38.8 | POS | 34.0 | POS |
| Sample 67 | MS2 | 25.9 |  | 27.0 |  | 26.0 |  |
| Sample 67 | N gene | 28.1 |  | 28.0 |  | 27.4 |  |
| Sample 67 | ORF1ab | 28.7 |  | 28.2 |  | 27.9 |  |
| Sample 67 | S gene | 28.1 | POS | 28.4 | POS | 26.3 | POS |
| Sample 68 | MS2 | 26.3 |  | 26.9 |  | 25.5 |  |
| Sample 68 | N gene | 26.7 |  | 26.6 |  | 26.2 |  |
| Sample 68 | ORF1ab | 27.4 |  | 27.0 |  | 26.3 |  |
| Sample 68 | S gene | 26.9 | POS | 27.3 | POS | 24.8 | POS |
| Sample 69 | MS2 | 27.1 |  | 27.0 |  | 25.9 |  |
| Sample 69 | N gene | 28.3 |  | 29.3 |  | 31.7 |  |
| Sample 69 | ORF1ab | 29.6 |  | 29.9 |  | 32.9 |  |
| Sample 69 | S gene | 29.0 | POS | 30.3 | POS | 31.3 | POS |
| Sample 70 | MS2 | 27.3 |  | 27.4 |  | 26.2 |  |
| Sample 70 | N gene | 24.6 |  | 25.6 |  | 26.8 |  |
| Sample 70 | ORF1ab | 25.5 |  | 25.7 |  | 28.4 |  |
| Sample 70 | S gene | 24.5 | POS | 25.8 | POS | 27.2 | POS |
| Sample 71 | MS2 | 26.8 |  | 27.4 |  | 25.3 |  |
| Sample 71 | N gene | 34.9 |  | 35.6 |  | 34.8 |  |
| Sample 71 | ORF1ab | 38.3 |  | UD |  | 33.9 |  |
| Sample 71 | S gene | UD | REP | 36.5 | POS | 32.1 | POS |
| Sample 72 | MS2 | 26.9 |  | 27.0 |  | 26.1 |  |
| Sample 72 | N gene | UD |  | UD |  | UD |  |
| Sample 72 | ORF1ab | UD |  | UD |  | UD |  |
| Sample 72 | S gene | UD | NEG | UD | NEG | UD | NEG |
| Sample 73 | MS2 | 27.7 |  | 27.6 |  | 25.6 |  |
| Sample 73 | N gene | 20.1 |  | 22.0 |  | 22.0 |  |
| Sample 73 | ORF1ab | 20.8 |  | 22.1 |  | 22.3 |  |
| Sample 73 | S gene | 20.8 | POS | 22.8 | POS | 20.9 | POS |
| Sample 74 | MS2 | 33.4 |  | 31.8 |  | 28.2 |  |
| Sample 74 | N gene | 17.1 |  | 18.5 |  | 17.8 |  |
| Sample 74 | ORF1ab | 18.0 |  | 19.0 |  | 18.2 |  |
| Sample 74 | S gene | 17.5 | POS | 19.6 | POS | 17.0 | POS |
| Sample 75 | MS2 | 26.0 |  | 27.0 |  | 25.2 |  |
| Sample 75 | N gene | UD |  | 32.8 |  | 30.3 |  |
| Sample 75 | ORF1ab | UD |  | 33.2 |  | 31.7 |  |
| Sample 75 | S gene | UD | NEG | 35.1 | POS | 29.8 | POS |
| Sample 76 | MS2 | 26.0 |  | 27.1 |  | 25.2 |  |
| Sample 76 | N gene | UD |  | UD |  | UD |  |
| Sample 76 | ORF1ab | UD |  | UD |  | UD |  |
| Sample 76 | S gene | UD | NEG | UD | NEG | UD | NEG |
| Sample 77 | MS2 | 25.9 |  | 26.8 |  | 24.9 |  |
| Sample 77 | N gene | UD |  | 39.9 |  | 36.8 |  |
| Sample 77 | ORF1ab | UD |  | UD |  | 35.6 |  |
| Sample 77 | S gene | UD | NEG | UD | NEG | 33.7 | POS |
| Sample 78 | MS2 | 26.6 |  | 26.7 |  | 25.1 |  |
| Sample 78 | N gene | UD |  | 38.3 |  | 39.7 |  |
| Sample 78 | ORF1ab | UD |  | UD |  | UD |  |
| Sample 78 | S gene | UD | NEG | UD | NEG | 39.2 | NEG |
| Sample 79 | MS2 | 25.9 |  | 26.7 |  | 25.0 |  |
| Sample 79 | N gene | UD |  | UD |  | 37.3 |  |
| Sample 79 | ORF1ab | UD |  | UD |  | UD |  |
| Sample 79 | S gene | UD | NEG | 39.0 | NEG | 39.7 | NEG |
| Sample 80 | MS2 | 26.2 |  | 26.6 |  | 25.5 |  |
| Sample 80 | N gene | UD |  | UD |  | UD |  |
| Sample 80 | ORF1ab | UD |  | UD |  | UD |  |
| Sample 80 | S gene | UD | NEG | 37.6 | NEG | UD | NEG |
| Sample 81 | MS2 | 26.4 |  | 26.9 |  | 25.1 |  |
| Sample 81 | N gene | UD |  | UD |  | UD |  |
| Sample 81 | ORF1ab | UD |  | UD |  | UD |  |
| Sample 81 | S gene | UD | NEG | UD | NEG | UD | NEG |
| Sample 82 | MS2 | 26.5 |  | 26.8 |  | 25.4 |  |
| Sample 82 | N gene | UD |  | UD |  | UD |  |
| Sample 82 | ORF1ab | UD |  | UD |  | UD |  |
| Sample 82 | S gene | UD | NEG | UD | NEG | UD | NEG |
| Sample 83 | MS2 | 26.9 |  | 26.8 |  | 25.5 |  |
| Sample 83 | N gene | UD |  | UD |  | UD |  |
| Sample 83 | ORF1ab | UD |  | UD |  | UD |  |
| Sample 83 | S gene | UD | NEG | UD | NEG | UD | NEG |
| Sample 84 | MS2 | 26.9 |  | 26.7 |  | 25.4 |  |
| Sample 84 | N gene | UD |  | UD |  | UD |  |
| Sample 84 | ORF1ab | UD |  | UD |  | UD |  |
| Sample 84 | S gene | UD | NEG | UD | NEG | UD | NEG |
| Sample 85 | MS2 | 26.4 |  | 26.9 |  | 25.5 |  |
| Sample 85 | N gene | UD |  | 36.4 |  | 33.1 |  |
| Sample 85 | ORF1ab | UD |  | 34.2 |  | 36.0 |  |
| Sample 85 | S gene | UD | NEG | 36.3 | POS | 32.6 | POS |
| Sample 86 | MS2 | 26.3 |  | 26.8 |  | 25.1 |  |
| Sample 86 | N gene | UD |  | UD |  | 39.0 |  |
| Sample 86 | ORF1ab | UD |  | UD |  | UD |  |
| Sample 86 | S gene | UD | NEG | UD | NEG | UD | NEG |
| Sample 87 | MS2 | 26.0 |  | 26.5 |  | 25.0 |  |
| Sample 87 | N gene | UD |  | UD |  | UD |  |
| Sample 87 | ORF1ab | UD |  | UD |  | UD |  |
| Sample 87 | S gene | UD | NEG | UD | NEG | UD | NEG |
| Sample 88 | MS2 | 26.2 |  | 26.5 |  | 24.9 |  |
| Sample 88 | N gene | UD |  | UD |  | UD |  |
| Sample 88 | ORF1ab | UD |  | UD |  | UD |  |
| Sample 88 | S gene | UD | NEG | UD | NEG | UD | NEG |
| Sample 89 | MS2 | 25.7 |  | 26.2 |  | 24.7 |  |
| Sample 89 | N gene | UD |  | UD |  | 37.9 |  |
| Sample 89 | ORF1ab | UD |  | UD |  | UD |  |
| Sample 89 | S gene | UD | NEG | UD | NEG | UD | NEG |
| Sample 90 | MS2 | 25.9 |  | 26.3 |  | 24.3 |  |
| Sample 90 | N gene | UD |  | UD |  | UD |  |
| Sample 90 | ORF1ab | UD |  | UD |  | UD |  |
| Sample 90 | S gene | UD | NEG | UD | NEG | UD | NEG |
| Sample 91 | MS2 | 26.0 |  | 26.0 |  | 24.8 |  |
| Sample 91 | N gene | 39.9 |  | UD |  | UD |  |
| Sample 91 | ORF1ab | UD |  | UD |  | UD |  |
| Sample 91 | S gene | UD | NEG | UD | NEG | UD | NEG |
| Sample 92 | MS2 | 26.3 |  | 26.2 |  | 24.3 |  |
| Sample 92 | N gene | UD |  | UD |  | UD |  |
| Sample 92 | ORF1ab | UD |  | UD |  | UD |  |
| Sample 92 | S gene | UD | NEG | UD | NEG | UD | NEG |
| Sample 93 | MS2 | 36.9 |  | 26.2 |  | 24.5 |  |
| Sample 93 | N gene | UD |  | UD |  | UD |  |
| Sample 93 | ORF1ab | UD |  | UD |  | UD |  |
| Sample 93 | S gene | UD | NEG | UD | NEG | 38.6 | NEG |
| Sample 94 | MS2 | 31.1 |  | 26.3 |  | 25.1 |  |
| Sample 94 | N gene | UD |  | UD |  | UD |  |
| Sample 94 | ORF1ab | 39.5 |  | UD |  | UD |  |
| Sample 94 | S gene | UD | NEG | UD | NEG | UD | NEG |
| POS | MS2 | UD |  | 30.0 |  | 31.3 |  |
| POS | N gene | 26.6 |  | 26.5 |  | 26.2 |  |
| POS | ORF1ab | 26.9 |  | 26.0 |  | 26.4 |  |
| POS | S gene | 26.4 |  | 26.4 |  | 25.1 |  |
| NEG | MS2 | 27.3 |  | 26.3 |  | 26.7 |  |
| NEG | N gene | UD |  | UD |  | 38.9 |  |
| NEG | ORF1ab | UD |  | UD |  | UD |  |
| NEG | S gene | UD |  | UD |  | UD |  |

**Supplementary Material 3. COVID-19 reference diagnosis and robotic MAVRICS extraction/R3T qRT-PCR results.** RNA extraction was performed on 106 clinical samples using the Tecan EVO-200 system. One-step RT-qPCR was performed with the R3T qRT-PCR system and the primers/probes nCoV_N1, nCoV_N2, and RNase P. The COVID-19 diagnosis of the samples was compared to the reference result (green for concordant result, and yellow for non-concordant result). The table shows the Ct values obtained by our platform.

|  |  | **SiMNP/Robotic extraction/R3T RT-qPCR** | | | |
| --- | --- | --- | --- | --- | --- |
| SAMPLE | **REFERENCE DIAGNOSIS** | **RESULT**  **IN THIS STUDY** | **RNAse P (Ct)** | **nCoV_N1 (Ct)** | **nCoV_N2 (Ct)** |
| 1 | POSITIVE | POSITIVE | 22.13 | 23.93 | 27.36 |
| 2 | POSITIVE | POSITIVE | 23.31 | 25.34 | 28.32 |
| 3 | NEGATIVE | NEGATIVE | 22.49 | No detected | No detected |
| 4 | NEGATIVE | NEGATIVE | 24.09 | No detected | No detected |
| 5 | NEGATIVE | NEGATIVE | 24.53 | No detected | No detected |
| 6 | NEGATIVE | NEGATIVE | 27.78 | No detected | No detected |
| 7 | NEGATIVE | NEGATIVE | 23.44 | No detected | No detected |
| 8 | NEGATIVE | NEGATIVE | 24.04 | No detected | No detected |
| 9 | NEGATIVE | NEGATIVE | 25.54 | No detected | No detected |
| 10 | NEGATIVE | NEGATIVE | 24.87 | No detected | No detected |
| 11 | NEGATIVE | NEGATIVE | 24.83 | No detected | No detected |
| 12 | NEGATIVE | NEGATIVE | 26.46 | No detected | No detected |
| 13 | NEGATIVE | NEGATIVE | 25.55 | No detected | No detected |
| 14 | NEGATIVE | NEGATIVE | 30.65 | No detected | No detected |
| 15 | NEGATIVE | NEGATIVE | 28.60 | No detected | No detected |
| 16 | NEGATIVE | NEGATIVE | 23.51 | No detected | No detected |
| 17 | NEGATIVE | NEGATIVE | 24.05 | No detected | No detected |
| 18 | NEGATIVE | NEGATIVE | 25.25 | No detected | No detected |
| 19 | NEGATIVE | NEGATIVE | 24.70 | No detected | No detected |
| 20 | NEGATIVE | NEGATIVE | 26.01 | No detected | No detected |
| 21 | NEGATIVE | NEGATIVE | 22.75 | No detected | No detected |
| 22 | NEGATIVE | NEGATIVE | 24.94 | No detected | No detected |
| 23 | NEGATIVE | NEGATIVE | 27.44 | No detected | No detected |
| 24 | NEGATIVE | NEGATIVE | 23.93 | No detected | No detected |
| 25 | NEGATIVE | NEGATIVE | 25.27 | No detected | No detected |
| 26 | NEGATIVE | NEGATIVE | 23.76 | No detected | No detected |
| 27 | NEGATIVE | NEGATIVE | 24.21 | No detected | No detected |
| 28 | NEGATIVE | NEGATIVE | 23.06 | No detected | No detected |
| 29 | NEGATIVE | NEGATIVE | 26.37 | No detected | No detected |
| 30 | NEGATIVE | NEGATIVE | 24.45 | No detected | No detected |
| 31 | NEGATIVE | NEGATIVE | 27.13 | No detected | No detected |
| 32 | NEGATIVE | NEGATIVE | 22.42 | No detected | No detected |
| 33 | NEGATIVE | NEGATIVE | 25.98 | No detected | No detected |
| 34 | NEGATIVE | NEGATIVE | 23.14 | No detected | No detected |
| 35 | NEGATIVE | NEGATIVE | 23.15 | No detected | No detected |
| 36 | NEGATIVE | NEGATIVE | 22.49 | No detected | No detected |
| 37 | POSITIVE | POSITIVE | 21.79 | 29.82 | 31.70 |
| 38 | POSITIVE | POSITIVE | 23.80 | 26.43 | 28.98 |
| 39 | POSITIVE | POSITIVE | 26.54 | 31.02 | 32.26 |
| 40 | POSITIVE | INVALID | No detected | No detected | No detected |
| 41 | POSITIVE | POSITIVE | 23.84 | 31.06 | 35.20 |
| 42 | POSITIVE | POSITIVE | 24.06 | 28.55 | 32.57 |
| 43 | POSITIVE | NEGATIVE | 22.53 | No detected | No detected |
| 44 | POSITIVE | POSITIVE | 24.46 | 29.87 | 30.94 |
| 45 | POSITIVE | POSITIVE | 28.52 | 35.33 | 35.33 |
| 46 | NEGATIVE | NEGATIVE | 25.47 | No detected | No detected |
| 47 | NEGATIVE | INCONCLUSIVE | 26.65 | 39.06 | Undetermined |
| 48 | NEGATIVE | NEGATIVE | 29.07 | Undetermined | Undetermined |
| 49 | NEGATIVE | INCONCLUSIVE | 27.00 | Undetermined | 37.56 |
| 50 | NEGATIVE | NEGATIVE | 25.04 | No detected | No detected |
| 51 | NEGATIVE | NEGATIVE | 24.35 | No detected | No detected |
| 52 | NEGATIVE | NEGATIVE | 23.78 | No detected | No detected |
| 53 | NEGATIVE | NEGATIVE | 28.75 | No detected | No detected |
| 54 | NEGATIVE | NEGATIVE | 23.40 | No detected | No detected |
| 55 | NEGATIVE | NEGATIVE | 25.05 | No detected | No detected |
| 56 | NEGATIVE | INCONCLUSIVE | 28.58 | No detected | 37.54 |
| 57 | NEGATIVE | NEGATIVE | 24.74 | No detected | No detected |
| 58 | NEGATIVE | NEGATIVE | 27.53 | No detected | 42.87 |
| 59 | NEGATIVE | NEGATIVE | 26.90 | No detected | No detected |
| 60 | INVALID | NEGATIVE | 35.22 | No detected | No detected |
| 61 | NEGATIVE | NEGATIVE | 27.11 | No detected | No detected |
| 62 | NEGATIVE | NEGATIVE | 25.13 | No detected | No detected |
| 63 | NEGATIVE | NEGATIVE | 28.04 | No detected | No detected |
| 64 | NEGATIVE | NEGATIVE | 26.47 | No detected | No detected |
| 65 | INCONCLUSIVE | NEGATIVE | 31.82 | No detected | No detected |
| 66 | INVALID | INVALID | Undetermined | No detected | No detected |
| 67 | NEGATIVE | NEGATIVE | 32.07 | No detected | No detected |
| 68 | INCONCLUSIVE | NEGATIVE | 34.33 | No detected | No detected |
| 69 | NEGATIVE | NEGATIVE | 30.64 | No detected | No detected |
| 70 | NEGATIVE | NEGATIVE | 37.29 | No detected | No detected |
| 71 | NEGATIVE | NEGATIVE | 32.04 | No detected | No detected |
| 72 | INCONCLUSIVE | NEGATIVE | 28.66 | No detected | No detected |
| 73 | NEGATIVE | NEGATIVE | 24.65 | No detected | No detected |
| 74 | INCONCLUSIVE | NEGATIVE | 28.51 | No detected | No detected |
| 75 | NEGATIVE | INCONCLUSIVE | 28.94 | No detected | 36.62 |
| 76 | NEGATIVE | NEGATIVE | 33.04 | No detected | No detected |
| 77 | NEGATIVE | INCONCLUSIVE | 32.59 | No detected | 37.45 |
| 78 | INCONCLUSIVE | NEGATIVE | 29.99 | No detected | 43.20 |
| 79 | INVALID | NEGATIVE | 32.66 | No detected | No detected |
| 80 | NEGATIVE | NEGATIVE | 28.66 | No detected | No detected |
| 81 | NEGATIVE | NEGATIVE | 24.42 | No detected | No detected |
| 82 | NEGATIVE | NEGATIVE | 37.13 | No detected | No detected |
| 83 | NEGATIVE | NEGATIVE | 26.97 | No detected | No detected |
| 84 | INVALID | INVALID | Undetermined | No detected | No detected |
| 85 | INCONCLUSIVE | INVALID | Undetermined | No detected | No detected |
| 86 | NEGATIVE | NEGATIVE | 25.72 | No detected | No detected |
| 87 | POSITIVE | INCONCLUSIVE | 21.90 | No detected | 38.04 |
| 88 | POSITIVE | POSITIVE | 22.29 | 29.19 | 32.26 |
| 89 | POSITIVE | POSITIVE | 22.45 | 31.06 | 35.01 |
| 90 | POSITIVE | POSITIVE | 25.14 | 33.64 | 35.02 |
| 91 | POSITIVE | POSITIVE | 26.07 | 22.78 | 25.36 |
| 92 | POSITIVE | POSITIVE | 22.26 | 28.79 | 32.00 |
| 93 | POSITIVE | POSITIVE | 24.45 | 29.63 | 32.17 |
| 94 | POSITIVE | POSITIVE | 23.07 | 33.63 | 38.03 |
| 95 | POSITIVE | POSITIVE | 23.43 | 33.91 | 37.09 |
| 96 | NEGATIVE | NEGATIVE | 26.63 | No detected | No detected |
| 97 | NEGATIVE | NEGATIVE | 26.72 | No detected | No detected |
| 98 | POSITIVE | POSITIVE | 24.72 | 37.7 | 35.61 |
| 99 | POSITIVE | NEGATIVE | 30.36 | No detected | 41.04 |
| 100 | POSITIVE | POSITIVE | 23.42 | 17.6 | 18.28 |
| 101 | POSITIVE | INCONCLUSIVE | 31.1 | 44.55 | 37.13 |
| 102 | POSITIVE | POSITIVE | 21.98 | 29.88 | 30.64 |
| 103 | POSITIVE | POSITIVE | 31.27 | 31.62 | 32.2 |
| 104 | POSITIVE | POSITIVE | 21.3 | 25.88 | 27.31 |
| 105 | POSITIVE | NEGATIVE | 23.89 | No detected | No detected |
| 106 | POSITIVE | POSITIVE | 26.17 | 27.65 | 29.34 |

**Supplementary material 4**. **Mutations present in SARS-CoV-2 variants covered by the five RPA amplicons**.

| **20I (Alpha, V1) (B.1.1.7)** | **20H (Beta, V2) (B.1.351)** | **20J (Gamma, V3) (P.1)** | **21A (Delta) (B.1.617.2)** | **21B (Kappa) (B.1.617.1)** | **21K (Omicron) (BA.1)** | **21L (Omicron) (BA.2)** | **22A & 22B (Omicron) (BA.4&5)** | **22C (Omicron) (BA.2.12.1)** | **22D (Omicron) (BA.2.75)** | **22E (Omicron) (BQ.1)** | **22F (Omicron) (XBB)** | **21D (Eta) (B.1.525)** | **21G (Lambda) (C.37)** |
| --- | --- | --- | --- | --- | --- | --- | --- | --- | --- | --- | --- | --- | --- |
| amplicon_5:34:A>G_D614G | amplicon_3:123_125:del_A243del | amplicon_2:25:G>T_D138Y | amplicon_5:34:A>G_D614G | amplicon_5:34:A>G_D614G | amplicon_1:176:C>T_A67V | amplicon_1:47_55:del_A27S | amplicon_1:47_55:del_A27S | amplicon_1:47_55:del_A27S | amplicon_1:47_55:del_A27S | amplicon_1:47_55:del_A27S | amplicon_1:47_55:del_A27S | amplicon_1:176:C>T_A67V | amplicon_3:150:G>A_D253N |
| amplicon_1:179_181:del_H69del | amplicon_3:37:A>G_D215G | amplicon_5:34:A>G_D614G | amplicon_2:79_81:del_E156del | amplicon_2:73:G>A_E154K | amplicon_5:34:A>G_D614G | amplicon_4:6:A>G_D405N | amplicon_4:6:A>G_D405N | amplicon_4:6:A>G_D405N | amplicon_4:6:A>G_D405N | amplicon_4:6:A>G_D405N | amplicon_4:6:A>G_D405N | amplicon_5:34:A>G_D614G | amplicon_5:34:A>G_D614G |
| amplicon_4:294:A>T_N501Y | amplicon_5:34:A>G_D614G | amplicon_4:243:G>A_E484K | amplicon_2:82_84:del_F157del | amplicon_4:243:G>C_E484Q | amplicon_4:244:A>C_E484A | amplicon_5:34:A>G_D614G | amplicon_5:34:A>G_D614G | amplicon_5:34:A>G_D614G | amplicon_5:34:A>G_D614G | amplicon_5:34:A>G_D614G | amplicon_5:34:A>G_D614G | amplicon_4:243:G>A_E484K | amplicon_4:262:T>C_F490S |
| amplicon_5:235:C>A_P681H | amplicon_1:215:A>C_D80A | amplicon_5:156:C>T_H655Y | amplicon_2:38:G>A_G142D | amplicon_4:148:T>G_L452R | amplicon_2:37_39:del_G142del | amplicon_4:244:A>C_E484A | amplicon_4:244:A>C_E484A | amplicon_4:244:A>C_E484A | amplicon_4:244:A>C_E484A | amplicon_4:244:A>C_E484A | amplicon_4:244:A>C_E484A | amplicon_1:179_181:del_H69del | amplicon_3:147_149:del_G252del |
| amplicon_1:182_184:del_V70del | amplicon_4:243:G>A_E484K | amplicon_4:43:A>C_K417T | amplicon_4:148:T>G_L452R | amplicon_5:235:C>G_P681R | amplicon_4:129:G>A_G446S | amplicon_2:38:G>A_G142D | amplicon_4:249:T>G_F486V | amplicon_2:38:G>A_G142D | amplicon_2:84:C>A_F157L | amplicon_4:249:T>G_F486V | amplicon_4:250:T>C_F486S | amplicon_1:131:A>G_Q52R | amplicon_1:200:G>T_G75V |
| amplicon_2:42_44:del_Y144del | amplicon_4:44:G>T_K417N | amplicon_1:28:C>T_L18F | amplicon_5:235:C>G_P681R |  | amplicon_4:279:G>A_G496S | amplicon_5:156:C>T_H655Y | amplicon_2:38:G>A_G142D | amplicon_5:156:C>T_H655Y | amplicon_2:38:G>A_G142D | amplicon_2:38:G>A_G142D | amplicon_4:262:T>C_F490S | amplicon_5:224:G>C_Q677H | amplicon_3:138_140:del_L249del |
|  | amplicon_1:28:C>T_L18F | amplicon_4:294:A>T_N501Y | amplicon_2:85:A>G_R158G |  | amplicon_5:156:C>T_H655Y | amplicon_4:44:G>T_K417N | amplicon_5:156:C>T_H655Y | amplicon_4:44:G>T_K417N | amplicon_3:162:G>A_G257S | amplicon_5:156:C>T_H655Y | amplicon_2:38:G>A_G142D | amplicon_1:182_184:del_V70del | amplicon_4:148:T>A_L452Q |
|  | amplicon_3:117_119:del_L241del | amplicon_1:52:C>T_P26S | amplicon_1:32:C>G_T19R |  | amplicon_1:179_181:del_H69del | amplicon_1:47_49:del_L24del | amplicon_1:179_181:del_H69del | amplicon_1:47_49:del_L24del | amplicon_4:129:G>A_G446S | amplicon_1:179_181:del_H69del | amplicon_4:129:G>A_G446S | amplicon_2:42_44:del_Y144del | amplicon_3:144_146:del_P251del |
|  | amplicon_3:120_122:del_L242del | amplicon_2:183:G>T_R190S | amplicon_4:226:C>A_T478K |  | amplicon_4:44:G>T_K417N | amplicon_4:113:T>G_N440K | amplicon_4:44:G>T_K417N | amplicon_4:148:T>A_L452Q | amplicon_5:156:C>T_H655Y | amplicon_4:44:G>T_K417N | amplicon_2:51:C>A_H146Q |  | amplicon_3:129_131:del_R246del |
|  | amplicon_4:294:A>T_N501Y | amplicon_1:35:C>A_T20N |  |  | amplicon_3:25_27:del_L212I | amplicon_4:294:A>T_N501Y | amplicon_1:47_49:del_L24del | amplicon_4:113:T>G_N440K | amplicon_3:21:A>G_I210V | amplicon_4:124:A>C_K444T | amplicon_5:156:C>T_H655Y |  | amplicon_3:132_134:del_S247del |
|  |  |  |  |  | amplicon_3:24_26:del_N211del | amplicon_5:230:T>G_N679K | amplicon_4:148:T>G_L452R | amplicon_4:294:A>T_N501Y | amplicon_2:52:A>G_K147E | amplicon_1:47_49:del_L24del | amplicon_4:44:G>T_K417N |  | amplicon_3:141_143:del_T250del |
|  |  |  |  |  | amplicon_4:113:T>G_N440K | amplicon_1:50_52:del_P25del | amplicon_4:113:T>G_N440K | amplicon_5:230:T>G_N679K | amplicon_4:44:G>T_K417N | amplicon_4:148:T>G_L452R | amplicon_1:47_49:del_L24del |  | amplicon_1:203:C>T_T76I |
|  |  |  |  |  | amplicon_4:294:A>T_N501Y | amplicon_1:53_55:del_P26del | amplicon_4:294:A>T_N501Y | amplicon_1:50_52:del_P25del | amplicon_1:47_49:del_L24del | amplicon_4:113:T>G_N440K | amplicon_4:113:T>G_N440K |  | amplicon_3:135_137:del_Y248del |
|  |  |  |  |  | amplicon_5:230:T>G_N679K | amplicon_5:235:C>A_P681H | amplicon_5:230:T>G_N679K | amplicon_1:53_55:del_P26del | amplicon_4:113:T>G_N440K | amplicon_4:173:T>G_N460K | amplicon_4:173:T>G_N460K |  |  |
|  |  |  |  |  | amplicon_5:235:C>A_P681H | amplicon_4:271:A>G_Q493R | amplicon_1:50_52:del_P25del | amplicon_5:235:C>A_P681H | amplicon_4:173:T>G_N460K | amplicon_4:294:A>T_N501Y | amplicon_4:294:A>T_N501Y |  |  |
|  |  |  |  |  | amplicon_4:271:A>G_Q493R | amplicon_4:286:A>G_Q498R | amplicon_1:53_55:del_P26del | amplicon_4:271:A>G_Q493R | amplicon_4:294:A>T_N501Y | amplicon_5:230:T>G_N679K | amplicon_5:230:T>G_N679K |  |  |
|  |  |  |  |  | amplicon_4:286:A>G_Q498R | amplicon_4:17:A>T_R408S | amplicon_5:235:C>A_P681H | amplicon_4:286:A>G_Q498R | amplicon_5:230:T>G_N679K | amplicon_1:50_52:del_P25del | amplicon_1:50_52:del_P25del |  |  |
|  |  |  |  |  | amplicon_4:223:G>A_S477N | amplicon_4:223:G>A_S477N | amplicon_4:286:A>G_Q498R | amplicon_4:17:A>T_R408S | amplicon_1:50_52:del_P25del | amplicon_1:53_55:del_P26del | amplicon_1:53_55:del_P26del |  |  |
|  |  |  |  |  | amplicon_4:226:C>A_T478K | amplicon_1:32:C>T_T19I | amplicon_4:17:A>T_R408S | amplicon_4:223:G>A_S477N | amplicon_1:53_55:del_P26del | amplicon_5:235:C>A_P681H | amplicon_5:235:C>A_P681H |  |  |
|  |  |  |  |  | amplicon_1:260:C>T_T95I | amplicon_4:226:C>A_T478K | amplicon_4:223:G>A_S477N | amplicon_1:32:C>T_T19I | amplicon_5:235:C>A_P681H | amplicon_4:272:A>G_Q493Q | amplicon_2:160:C>G_Q183E |  |  |
|  |  |  |  |  | amplicon_2:40_42:del_V143del | amplicon_3:31:T>G_V213G | amplicon_1:32:C>T_T19I | amplicon_4:226:C>A_T478K | amplicon_4:286:A>G_Q498R | amplicon_4:286:A>G_Q498R | amplicon_4:286:A>G_Q498R |  |  |
|  |  |  |  |  | amplicon_1:182_184:del_V70del | amplicon_4:306:T>C_Y505H | amplicon_4:226:C>A_T478K | amplicon_3:31:T>G_V213G | amplicon_4:17:A>T_R408S | amplicon_4:17:A>T_R408S | amplicon_4:17:A>T_R408S |  |  |
|  |  |  |  |  | amplicon_2:42_44:del_Y144del |  | amplicon_3:31:T>G_V213G | amplicon_4:306:T>C_Y505H | amplicon_4:271:G>A_R493Q | amplicon_4:223:G>A_S477N | amplicon_4:271:G>A_R493Q |  |  |
|  |  |  |  |  | amplicon_2:46:T>G_Y145D |  | amplicon_1:182_184:del_V70del |  | amplicon_4:223:G>A_S477N | amplicon_1:32:C>T_T19I | amplicon_4:223:G>A_S477N |  |  |
|  |  |  |  |  | amplicon_4:306:T>C_Y505H |  | amplicon_4:306:T>C_Y505H |  | amplicon_1:32:C>T_T19I | amplicon_4:226:C>A_T478K | amplicon_1:32:C>T_T19I |  |  |
|  |  |  |  |  | amplicon_2:38_46:del_G142D/GVYdel |  |  |  | amplicon_4:226:C>A_T478K | amplicon_3:31:T>G_V213G | amplicon_4:226:C>A_T478K |  |  |
|  |  |  |  |  |  |  |  |  | amplicon_3:31:T>G_V213G | amplicon_1:182_184:del_V70del | amplicon_3:31:T>A_V213E |  |  |
|  |  |  |  |  |  |  |  |  | amplicon_2:67:T>C_W152R | amplicon_4:306:T>C_Y505H | amplicon_4:126_127:GT>CC_V445P |  |  |
|  |  |  |  |  |  |  |  |  | amplicon_4:306:T>C_Y505H |  | amplicon_1:224:T>C_V83A |  |  |
|  |  |  |  |  |  |  |  |  |  |  | amplicon_2:42_44:del_Y144del |  |  |
|  |  |  |  |  |  |  |  |  |  |  | amplicon_4:306:T>C_Y505H |  |  |

**Supplementary material 5. Sequencing reads and threshold criterion**. Number of reads in the sequenced samples and read count threshold criterion. Detection reads threshold was fixed as the highest number of reads in NTC samples (shading yellow reads) multiply by 2, the amplicons with number of reads below this threshold (red and cross out reads) were removed from further analysis.

| **#barcode** | **sample** | **read** | **nucleotide base number** | **amplicon_1** | **amplicon_2** | **amplicon_3** | **amplicon_4** | **amplicon_5** | **ACTB_263bp** | **NIRVANA result** |
| --- | --- | --- | --- | --- | --- | --- | --- | --- | --- | --- |
| barcode09 | NTC-R1 | 34,724 | 9,542,881 | ~~0~~ | ~~0~~ | ~~1~~ | ~~4~~ | ~~0~~ | ~~0~~ | UNKNOW |
| barcode05 | NTC-R2 | 41,121 | 12,231,264 | ~~0~~ | ~~0~~ | ~~1~~ | ~~9~~ | ~~2~~ | ~~3~~ | UNKNOW |
| barcode06 | NTC-R3 | 36,726 | 8,991,472 | ~~0~~ | ~~0~~ | ~~5~~ | ~~9~~ | ~~3~~ | ~~1~~ | UNKNOW |
| barcode10 | NTC-R4 | 36,180 | 9,815,164 | ~~0~~ | ~~0~~ | ~~2~~ | ~~3~~ | ~~0~~ | ~~0~~ | UNKNOW |
| barcode01 | NTC-R5 | 33,210 | 11,684,232 | ~~0~~ | ~~0~~ | ~~1~~ | ~~0~~ | ~~0~~ | ~~0~~ | UNKNOW |
| barcode02 | NTC-R6 | 68,303 | 24,880,434 | ~~0~~ | ~~1~~ | ~~2~~ | ~~1~~ | ~~0~~ | ~~0~~ | UNKNOW |
| barcode22 | NTC-RT1 | 44,637 | 13,171,925 | ~~0~~ | ~~2~~ | ~~7~~ | ~~4~~ | ~~1~~ | ~~1~~ | UNKNOW |
| barcode01 | NTC-RPA1 | 40,222 | 15,407,348 | ~~0~~ | ~~0~~ | ~~0~~ | ~~0~~ | ~~0~~ | ~~0~~ | UNKNOW |
| barcode13 | NTC-RPA2 | 30,907 | 11,897,873 | ~~0~~ | ~~0~~ | ~~0~~ | ~~0~~ | ~~0~~ | ~~0~~ | UNKNOW |
| barcode18 | NTC-RPA3 | 28,783 | 8,010,842 | ~~1~~ | ~~0~~ | ~~0~~ | ~~0~~ | ~~2~~ | ~~1~~ | UNKNOW |
| barcode19 | NTC-RPA4 | 34,005 | 8,311,274 | ~~0~~ | ~~1~~ | ~~1~~ | ~~7~~ | ~~1~~ | ~~1~~ | UNKNOW |
| barcode03 | NTC-RPA5 | 90,157 | 30,349,327 | ~~1~~ | ~~1~~ | ~~1~~ | ~~4~~ | ~~2~~ | ~~0~~ | UNKNOW |
| barcode04 | NTC-RPA6 | 43,716 | 15,074,614 | ~~2~~ | ~~2~~ | ~~0~~ | ~~0~~ | ~~0~~ | ~~0~~ | UNKNOW |
|  |  |  |  |  |  |  |  |  |  |  |
| barcode07 | NEG. 1 | 25,831 | 7,254,222 | ~~0~~ | ~~0~~ | ~~0~~ | ~~1~~ | ~~0~~ | 5,773 | NEGATIVE |
| barcode08 | NEG. 2 | 69,152 | 17,636,290 | ~~0~~ | ~~0~~ | ~~1~~ | ~~4~~ | ~~0~~ | 9,612 | NEGATIVE |
| barcode11 | NEG. 3 | 39,575 | 11,027,231 | ~~0~~ | ~~1~~ | ~~4~~ | ~~4~~ | ~~0~~ | 12,196 | NEGATIVE |
| barcode05 | NEG. 4 | 83,818 | 30,816,432 | ~~0~~ | ~~0~~ | ~~2~~ | ~~0~~ | ~~2~~ | 6,413 | NEGATIVE |
| barcode06 | NEG. 5 | 83,094 | 30,927,626 | ~~0~~ | ~~0~~ | ~~1~~ | ~~0~~ | ~~1~~ | 3,539 | NEGATIVE |
|  |  |  |  |  |  |  |  |  |  |  |
| barcode86 | POS. 1 | 31,528 | 10,481,565 | ~~0~~ | 75 | 674 | 437 | ~~1~~ | 868 | POSITIVE |
| barcode14 | POS. 2 | 73,294 | 22,015,121 | 56 | 388 | 7,336 | 2,398 | 149 | 2,861 | POSITIVE |
| barcode27 | POS. 3 | 36,546 | 10,693,062 | ~~0~~ | 13 | 1,594 | 37 | 314 | 6,782 | POSITIVE |
| barcode50 | POS. 4 | 71,187 | 23,788,710 | 461 | 5,038 | 8,651 | 12,654 | 381 | 3,377 | POSITIVE |
| barcode63 | POS. 5A | 82,050 | 25,390,830 | 483 | 7,533 | 12,431 | 20,087 | 5,209 | 2,422 | POSITIVE |
| barcode12 | POS. 5B | 95,077 | 28,480,536 | 1,542 | 8,281 | 22,781 | 23,812 | 13,444 | 3,250 | POSITIVE |
| barcode74 | POS. 6 | 55,333 | 16,149,968 | 7 | 144 | 1,606 | 899 | 522 | 9,057 | POSITIVE |
| barcode75 | POS. 7 | 49,536 | 15,053,660 | 40 | 22 | 2,479 | 723 | 301 | 11,144 | POSITIVE |
| barcode87 | POS. 8 | 40,756 | 11,547,155 | 6 | 449 | 5,312 | 153 | 19 | 4,907 | POSITIVE |
| barcode08 | POS. 9 | 86,916 | 29,444,887 | ~~2~~ | 5,425 | 10,559 | 17,818 | 11,910 | 4,070 | POSITIVE |
| barcode10 | POS. 10 | 100,369 | 37,110,270 | 7,912 | 21,567 | 17,370 | 17,204 | 14,677 | 308 | POSITIVE |
| barcode11 | POS. 11 | 86,139 | 28,590,522 | 5 | 7 | ~~2~~ | ~~2~~ | ~~3~~ | 420 | POSITIVE |
| barcode13 | POS. 12 | 87,844 | 29,879,627 | 232 | 4,707 | 7,603 | 7,198 | 5,785 | 697 | POSITIVE |
|  |  |  |  |  |  |  |  |  |  |  |
| unclassified99 |  | 63,718 | 55,648,809 | 890 | 3,454 | 3,699 | 5,480 | 3,649 | 1,592 |  |

**Supplementary material 6. Correlation between Ct values (nCoV_N1 and nCoV_N2) and sum reads for the five SARS-CoV-2 amplicons**.

**Supplementary Material 7. Raw gel pictures.**

**Related to Figure 4A**

**
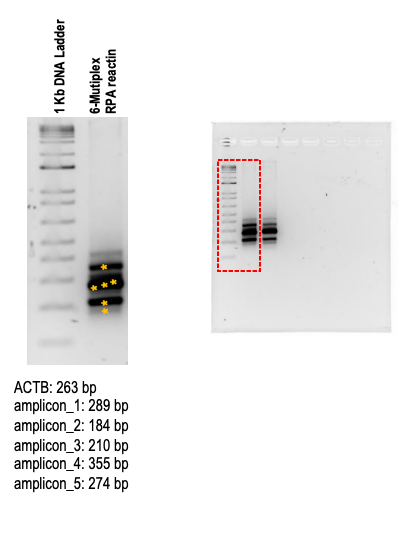
**

**Related to Supplementary Figure 3**

**
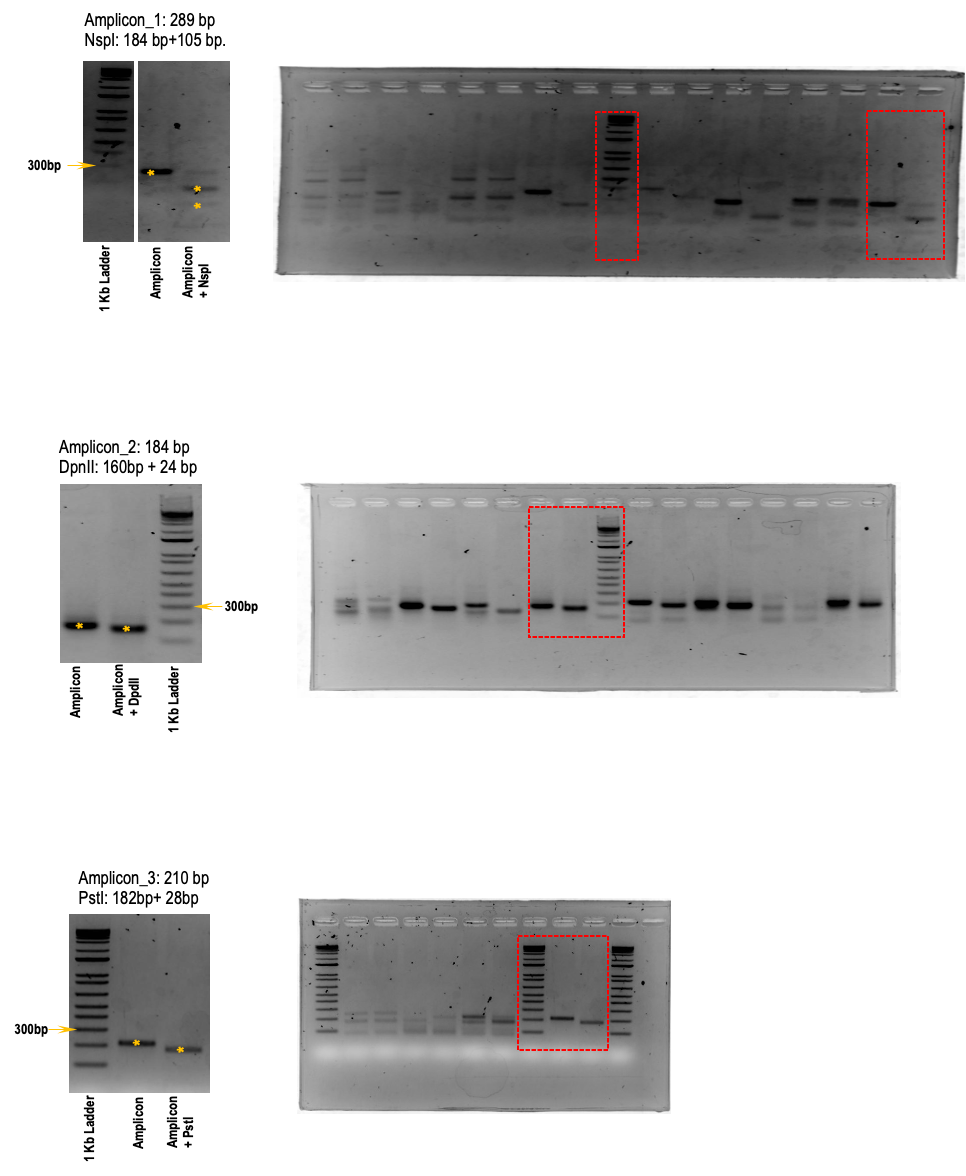
**

**Related to Supplementary Figure 3**


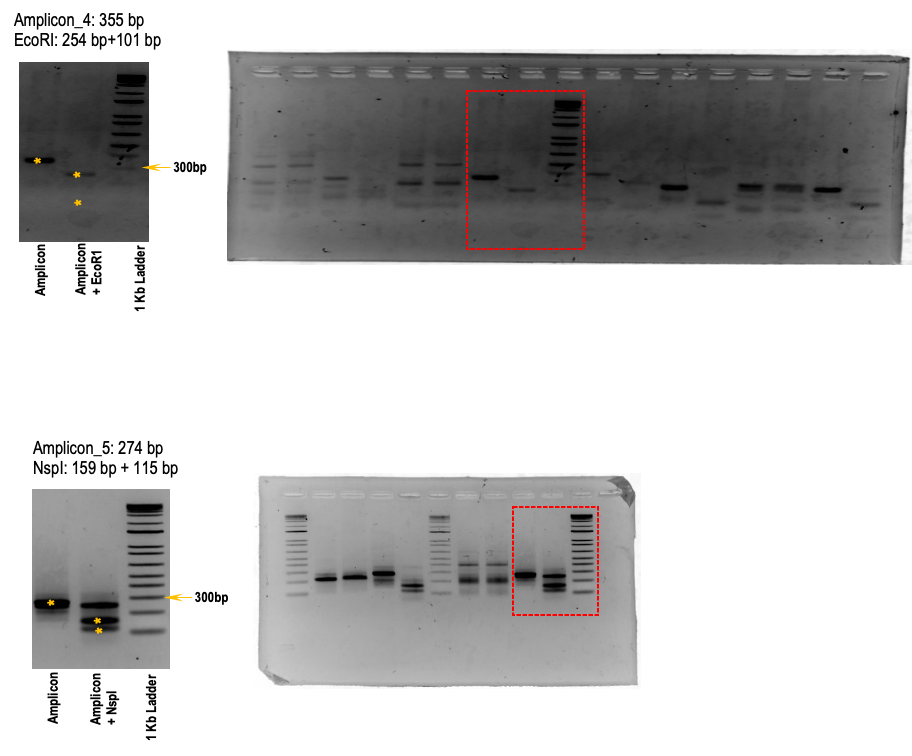

Supplement: Supplementary file 1 — Supplementary Information. [file 41598_2023_47190_MOESM1_ESM.docx]
